# Resolving the Evolutionary History of *Campanula* (Campanulaceae) in Western North America

**DOI:** 10.1371/journal.pone.0023559

**Published:** 2011-09-09

**Authors:** Barry M. Wendling, Kurt E. Galbreath, Eric G. DeChaine

**Affiliations:** Department of Biology, Western Washington University, Bellingham, Washington, United States of America; University of Otago, New Zealand

## Abstract

Recent phylogenetic works have begun to address long-standing questions regarding the systematics of *Campanula* (Campanulaceae). Yet, aspects of the evolutionary history, particularly in northwestern North America, remain unresolved. Thus, our primary goal in this study was to infer the phylogenetic positions of northwestern *Campanula* species within the greater Campanuloideae tree. We combined new sequence data from 5 markers (*atpB*, *rbcL*, *matK*, and *trnL-F* regions of the chloroplast and the nuclear ITS) representing 12 species of *Campanula* with previously published datasets for worldwide campanuloids, allowing us to include approximately 75% of North American *Campanuleae* in a phylogenetic analysis of the Campanuloideae. Because all but one of North American *Campanula* species are nested within a single campanuloid subclade (the *Rapunculus* clade), we conducted a separate set of analyses focused specifically on this group. Our findings show that i) the campanuloids have colonized North America at least 6 times, 4 of which led to radiations, ii) all but one North American campanuloid are nested within the *Rapunculus* clade, iii) in northwestern North America, a *C. piperi – C. lasiocarpa* ancestor gave rise to a monophyletic Cordilleran clade that is sister to a clade containing *C. rotundifolia*, iv) within the Cordilleran clade, *C. parryi* var. *parryi* and *C. parryi* var. *idahoensis* exhibit a deep, species-level genetic divergence, and v) *C. rotundifolia* is genetically diverse across its range and polyphyletic. Potential causes of diversification and endemism in northwestern North America are discussed.

## Introduction

The Campanulaceae, or Bellflowers, is a nearly cosmopolitan family known worldwide for its attractive bluish, bell-shaped flowers. Though it has received considerable attention by taxonomists, the classification and phylogenetic relationships within the family remain highly controversial. The family has a complex biogeographic history marked by episodes of rapid diversification [1]. The 84 genera of Campanulaceae are divided among five subfamilies, the largest and most widespread being Campanuloideae Burnett and Lobelioideae Burnett, with 1045 and 1192 species, respectively [2]. However, the numbers of constituent taxa vary widely according to different authorities, likely due to the uncertain phylogeny of the group. Within the Campanuloideae, taxonomists variously assign species to one of 35 to 55 genera across three tribes – the Campanuleae, the Wahlenbergiaeae, and the Platycodoneae [3], [4]. Most species (ca. 420) are currently included within the poorly resolved, polyphyletic genus *Campanula* L. [2], [3], [5] of the tribe Campanuleae [6].

*Campanula* are mostly herbaceous with pentamerous flowers and usually display a campanulate, infundibuliform, tubular, or rotate corolla [7], [8]. The majority of species exhibit a circum-Mediterranean distribution, ranging from the Arctic and north temperate zones to eastern Africa, southern Asia, and northern Mexico [2]. Across those regions, species inhabit meadows, woodland-edges, moorlands, cliffs, steppes, and tundra [9], [10]. Though the genus *Campanula* has a long history of taxonomic inquiry [11], [12], there is no consensus for infrageneric classifications, due in part to low taxonomic sampling, geographic biases, and a limited number of characters being incorporated into the analyses [1], [2], [13]. Yet, recent phylogenetic studies have provided considerable insights and demonstrate support for two major clades within *Campanula*: *Campanula sensu stricto* (s. str.) and *Rapunculus* [1], [3], [4], [14], [15]. The *Campanula* s. str. clade is a well-supported, diverse assemblage of European, north African, and Macronesian *Campanula* species, *Azorina* Feer from the Azores Islands, and *Feeria* Buser from Morocco. The highly supported *Rapunculus* clade includes species from east Asian, Mediterranean, and North American *Campanula* as well as taxa from 11 other smaller genera that lack calyx appendages and have a wide range of floral forms [1], [3].

While several family-wide and geographically broad phylogenetic analyses have helped to identify and clarify relationships among campanuloid clades [1], [3], [4], [14]–[16], these studies have incorporated different taxa and various genetic markers, limiting their utility in developing a single comprehensive phylogeny for the group. Further, to date, only about one third of North American *Campanula* species have been included in phylogenetic analyses. Speculation on the relationships among the North American species consists largely of intuitive classifications, anecdotal observations [17], and relationships implied by regional floras [18]. For example, upon discovering the original type specimen of *Campanula piperi* Howell, Piper [19] initially mis-identified the plant as *C. aurita* Greene, which may have led to the view that *C. piperi* and *C. aurita* are closely related [20].

*Campanula* is the largest of the 6–7 genera of Campanuloideae in North America, with ca. 23 species [2], seventy-five percent of which are rare or endemic [21]. It is likely that *Campanula* colonized the continent multiple times by way of the Bering Land Bridge and/or north Atlantic routes during the mid to late Tertiary and Quaternary [14], [21]–[23], a scenario that is consistent with intercontinental range expansions of other northern plant species, such as members of *Dryas* L. [24], *Rhododendron* L. [25], and *Saxifraga* L. [26], [27]. Once on the continent, diversification and radiation of *Campanula* species ensued [14], [22].

The complex history of colonization and diversification of *Campanula* in North America remains a mystery. The most comprehensive sampling effort to date, which set out in part to investigate the origin and diversification of Campanulaceae on the continent, included a total of 14 native North American species representing the five campanuloid genera [3]. However, only ∼30% of the native North American *Campanula* species were included. Even with this sparse sampling, previously hypothesized radiations on the Pacific coast [22] were supported [3]. Most of the North American species were nested within the morphologically heterogeneous *Rapunculus* clade and their phylogenetic positions suggest three or more colonizations of North America [1], [3], [4], [16]. More specifically, Roquet et al. [4] inferred a recent arrival of *C. rotundifolia* into North America based on the species' origination in Eurasia during the Pleistocene (∼500-300 kya). Within the *Rapanculus* clade, two subclades are evident: 1) *Rapanculus* 1, which is predominantly made up of species from the central and southern European mountains, including a limited number of *Adenophora* Fisch. and members of *Campanula* in the subsection *Isophylla* Damboldt and the sections *Heterophylla* (Witas.) Fed. and *Rapanculus* (Fourr.) Boisier and 2) the geographically diverse *Rapunculus* 2 clade, which consists of *Campanula* species and several other genera, including *Asyneuma* Griseb. and Schenk, *Legousia* Durand, *Petromarula* Vent. ex Hedw. f., *Phyteuma* L., and *Triodanis* Raf. ex Greene ([4], but see [2]). Recent molecular analyses that have illuminated this structure (e.g., [3], [4]) provide a foundation for investigating the history and diversification of *Campanula* in North America.

We addressed two large gaps concerning the history and diversification of *Campanula* in North America. First, we set out to resolve the phylogenetic positions of northwestern North American *Campanula* by expanding the sample. Our study group included approximately 75% of the North American *Campanula*, 12 of which inhabit the northwest, in one phylogenetic analysis of the Campanuloideae. In doing so, we filled a considerable taxonomic hole in previous analyses, permitting a more robust estimate of the probable number of colonization events into North America and revealing new insights into patterns of radiation and endemism, particularly for northwestern taxa. Because most of the North American *Campanula* are nested within the *Rapunculus* clade, our second objective was to develop a well-resolved picture of relationships within that clade. To accomplish these goals, we performed phylogenetic analyses on 17 native *Campanula* from North America and all campanuloid species available in Genbank, employing the complete suite of markers used to date by other researchers (chloroplast [cpDNA] *trnL-F*, *matK*, *rbcL*, *atpB*, and the nuclear [nDNA] ITS).

## Results

**Figure 1:**
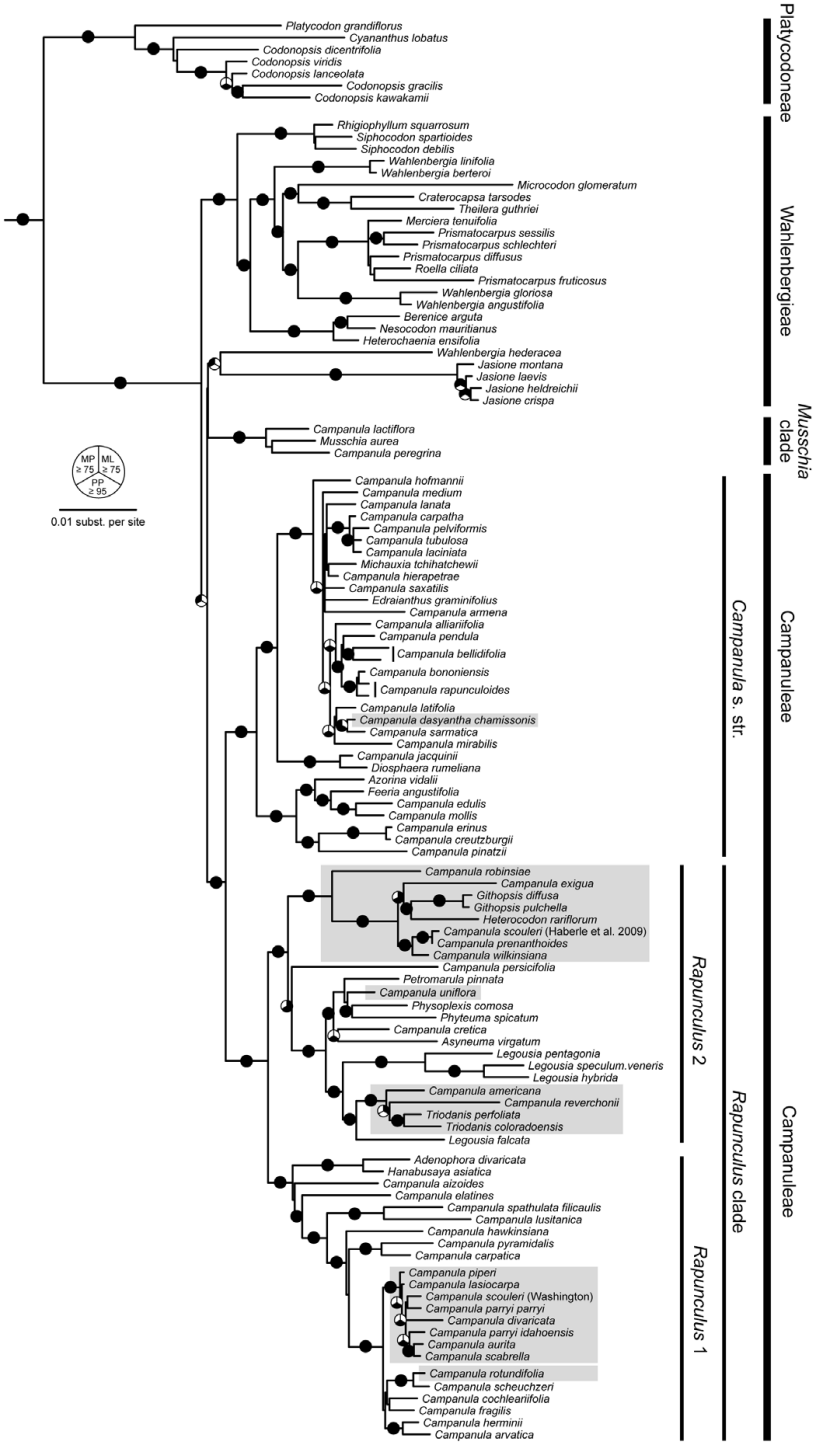
Best maximum likelihood phylogeny for the Campanulaceae based on concatenated *atpB*, *matK*, and *rbcL* DNA sequences. Outgroups have been removed for clarity. Pie graphs on branches indicate relationships that are well-supported under one or more of the three tree-building methods that we employed. Black-filled segments of the graphs indicate maximum parsimony (upper left) or maximum likelihood (upper right) bootstrap values ≥75, or Bayesian posterior probabilities ≥0.95 (bottom). Gray shading denotes North American species.

**Figure 2:**
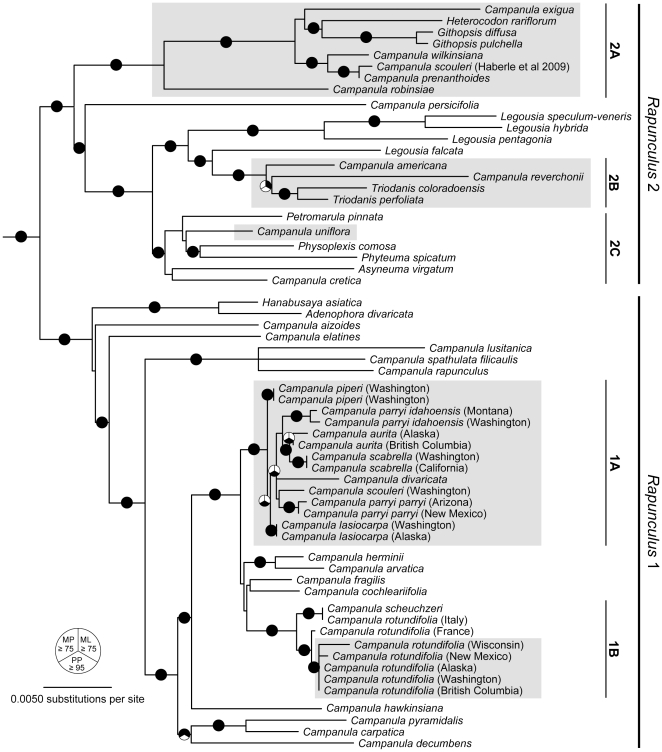
Best maximum likelihood phylogeny for the *Rapunculus* clade of the Campanuleae based on concatenated *atpB*, *matK*, *rbcL*, and *trnL-F* DNA sequences. Outgroups have been removed for clarity. Pie graphs on branches indicate relationships that are well-supported under one or more of the three tree-building methods that we employed. Black-filled segments of the graphs indicate maximum parsimony (upper left) or maximum likelihood (upper right) bootstrap values ≥75, or Bayesian posterior probabilities ≥0.95 (bottom). Gray shading denotes North American species. Subclades *Rapunculus* 1A, 1B, 2A, 2B, and 2C are discussed in the text.

Phylogenetic inferences were robust to tree-building methods, with Maximum Likelihood (ML), Maximum Parsimony (MP), and Bayesian approaches yielding concordant tree topologies. Trees based on individual cpDNA markers are largely consistent with one another, taking into account differences in taxon sampling within each dataset (Figs. S1, S2, S3, and S4). Instances of incongruence generally involve poorly supported relationships. Trees based on concatenated cpDNA datasets provide the best resolution and are sufficient to show key relationships between the Northwestern *Campanula* and the rest of the Campanulaceae. We therefore present the ML trees and associated nodal support values for two sets of concatenated analyses focused on broad (Campanulaceae-wide; Fig. 1) and narrow (*Rapunculus* clade only; Fig. 2) taxonomic scales (see Methods).

The cpDNA trees show that the North American *Campanula* are polyphyletic, appearing in at least six distinct clades within the Campanulaceae. *Campanula dasyantha* subsp. *chamissonis* (Fed.) Victorov is most divergent from the rest and is embedded in the *Campanula* s. str. clade, while *C. uniflora* L. is associated with the *Asyneuma*-*Phyteuma-Petromarula* alliance of *Rapunculus* 2 (Fig. 1). In contrast, most of the *Campanula* taxa from which we collected new data are clustered within the *Rapunculus* 1 group (Figs. 1, 2). *Campanula rotundifolia* L. is paraphyletic with respect to *C. scheuchzeri* Lodd., but together they form a strongly supported monophyletic clade associated with other European species (Fig. 2). The closest relatives of this Old World group form a monophyletic cluster of North American species that we refer to as the ‘Cordilleran’ clade due to its strong association with montane environments. Included in this clade are several taxa from northwestern North America (*C. piperi*, *C. lasiocarpa* Cham., *C. parryi* var. *idahoensis* McVaugh, *C. parryi* var. *parryi* A. Gray, *C. scabrella* Englem., *C. scouleri* Hook. ex A. DC.) as well as *C. divaricata* Michx. from eastern North America (but see ITS results below). Our conclusion that *C. scouleri* is a member of the Cordilleran clade contrasts with evidence reported by Haberle et al. [4] that placed the species in *Rapunculus* 2 associated with *C. prenanthoides* Durand (Fig. 2). While not fully resolved, certain structure within the Cordilleran clade is evident. Notably, *C. parryi* var. *idahoensis* and *C. parryi* var. *parryi* are not sister, but instead exhibit deep divergence consistent with species-level differences. Furthermore, *C. piperi* and *C. lasiocarpa* are basal to the other members of the clade.

**Figure 3:**
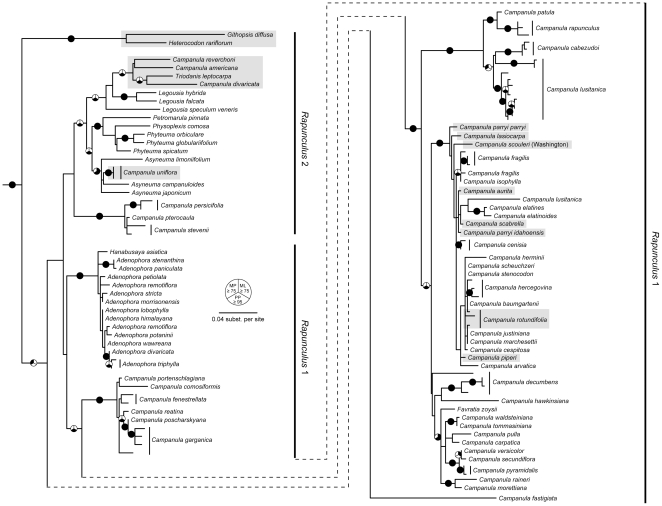
Best maximum likelihood phylogeny for the Campanulaceae based on ITS DNA sequences. Outgroups have been removed for clarity. Pie graphs on branches indicate relationships that are well-supported under one or more of the three tree-building methods that we employed. Black-filled segments of the graphs indicate maximum parsimony (upper left) or maximum likelihood (upper right) bootstrap values ≥75, or Bayesian posterior probabilities ≥0.95 (bottom). Maximum parsimony bootstrap values shown here are based on analyses that incorporated data from indels using simple indel coding (Simmons and Ochoterena 2000). Gray shading denotes North American species.

Phylogenetic results based on ITS are broadly consistent with that of the cpDNA analysis, though relationships within the ITS tree are generally less well supported (Fig. 3). In MP analyses, the use of simple indel coding (SIC) and modified complex indel coding (MCIC) schemes resulted in roughly equivalent improvements in bootstrap support for several nodes relative to simply removing indels prior to analysis. As in the cpDNA results, the ITS data retrieved a well-supported clade that included the majority of the Northwestern *Campanula* (excluding *C. dasyantha* subsp. *chamissonis* and *C. uniflora*) as well as several Old World species. However, there is essentially no resolution within this clade in the ITS tree, offering a poor test of relationships inferred from the cpDNA data. A notable difference between the cpDNA and nDNA trees is the position of *C. divaricata*. In contrast to the cpDNA tree, the ITS tree places this species firmly within *Rapunculus* 2 rather than *Rapunculus* 1.

## Discussion

The broad phylogenetic investigations into the Campanulaceae [1], [3]–[5], [14], [15], [28] set the stage for more focused biogeographic and taxonomic analyses [16]. Our analyses provide new insights into the biogeographic and evolutionary history of *Campanula* in North America. The history of *Campanula* in North America is marked by multiple (at least 6) colonization events that gave rise to several radiations, ultimately resulting in a diverse array of Campanuleae on the continent.

The Campanuloideae tree (Fig. 1) generated from three concatenated cpDNA markers exhibits a high degree of structure, revealing the diversity of North American *Campanula* and their evolutionary relationships. While it is not the purpose of this study to re-examine portions of pre-existing trees, for good interpretations of them are available elsewhere [1], [3], [4], several aspects are central to the systematics of the North American taxa. First, *Campanula* is polyphyletic and as this and other studies [3], [4], [5] show, taxonomic revisions at the generic level are warranted if taxonomy is to reflect phylogeny. Second, two major clades are evident, *Campanula* s. str. and *Rapunculus* [3], [4] following earlier taxonomic works by Boissier [12] and Fedorov [9], with the vast majority of North American taxa falling within the *Rapunculus* clade. The single exception, *C. dasyantha* subsp. *chamissonis*, nests within the *Campanula s. str*. clade, signifying the only colonization of North America (the Aleutian Islands) from a member of this clade. By contrast, our analyses suggest that members of the *Rapunculus* clade colonized North America at least five times, resulting in several radiations and comprising almost half of the diversity of this clade (at least among those that have been included in molecular analyses).

The *Rapunculus* clade exhibits a topological pattern of no fewer than 5 colonizations of North America and 4 radiations on the continent, and is partitioned into two distinct clades (*Rapunculus* 1 and *Rapunculus* 2, following Roquet and colleagues [4]) that differ dramatically in the geographic distribution and generic classification of their constituent taxa. Within these clades we highlight 5 subclades in the cpDNA tree (*Rapunculus* 1A and 1B; *Rapunculus* 2A, 2B, and 2C; see Fig. 2) that represent the smallest strongly supported clusters that were involved in each of the North American colonization events.

The *Rapunculus* 1 clade consists primarily of perennial *Campanula*, but also includes species of *Hanabusaya* Nakai and *Adenophora*. In both cpDNA and ITS phylogenies, there is strong support indicating that these genera are sister to the *Campanula* clade (Figs. 2, 3). Eddie [29] suggested that more recent invasions into North America may have occurred by members of the subsection *Heterophylla*, which includes *C. rotundifolia*, and our data are consistent with this hypothesis. Two subclades within *Rapunculus* 1 (1A, 1B) include North American taxa and presumably reflect independent dispersal events into the New World. Apparent incongruence in the ITS tree appears to be due to overall poorly resolved relationships at shallow scales, which preclude strong inferences based on the nDNA locus. Nearly all the 1A and 1B *Campanula* occur in the mountain ranges of western North America, though their distributions range from circumboreal to highly restricted.

*Campanula rotundifolia* has been included in most phylogenetic analyses of *Campanula*, but the cohesiveness of the species remains uncertain [2] given high phenotypic variation across its widespread distribution. Thus, we sampled *C. rotundifolia* across North America and Europe to determine whether the taxon is monophyletic [2] and to understand its role in the origin and diversification of North American *Campanula*. Our analyses show that *C. rotundifolia* is paraphyletic with respect to *C. scheuchzeri*, and that North American populations form a monophyletic clade that is nested within those from the Old World (Fig. 2). The species evidently colonized North America in a single event from European ancestors. Subsequently, North American *C. rotundifolia* diverged into separate morphotypes [30] that may represent distinct species [2]. Our broad geographic sampling within North America revealed genetic variation across the region, but additional molecular phylogeographic analyses with better geographic sampling across the species' circumboreal range will be necessary to resolve patterns of diversity in this putative species complex [2].

The strongly-supported *Rapunculus* 1A (Cordilleran) clade is central to understanding the evolutionary history of the majority of the diversity of *Campanula* in northwestern North America. All but one member of this clade (*C. lasiocarpa*) are endemic to North America, apparently arising during a radiation that followed a single colonization. This colonization is separate from that of *C. rotundifolia*, and the distinction between these two groups is consistent with morphological differences. For example, members of *Rapunculus* 1A lack the dimorphic leaves and basal capsule pores evident in *C. rotundifolia.* Instead, the oldest lineages in the Cordilleran clade (*C. piperi* and *C. lasiocarpa*) share a distinct form of mucronate leaf margin. *Campanula piperi*, which is best-supported as the most basal taxon, is a narrow endemic of the subalpine-alpine environs of the Olympic Mountains of Washington state. In contrast, the present range of *C. lasiocarpa* overlaps with most other members of the group. It is also the only member of the clade with a distribution that spans Beringia (eastern Siberia and Alaska), the likely route of North American colonization.

Two varieties of *C. parryi* were examined in the study. The results show that *C. parryi* var. *parryi* and *C. parryi* var. *idahoensis* are not sisters and probably represent two distinct species with separate geographic ranges. Population distributions of *C. parryi* var. *idahoensis* and *C. parryi* var. *parryi* are delimited by three physiographic regions of the Rocky Mountains. The more diminutive *C. parryi* var. *idahoensis* inhabits high-elevation portions of the Northern Rockies and North Cascades mountain ranges, while *C. parryi* var. *parryi* is restricted to high-elevation areas of the Southern Rockies. Neither taxa are abundant in the intervening central Rockies or the Wyoming Basin which forms an ecological barrier for high-elevation adapted flora [31]. The present day floristic composition of the lower Northern Rockies shows strong affinities to the Pacific Northwest and represents an eastward extension of the Vancouverian floristic province [32], [33]. Although part of the Rocky Mountain Floristic Province, the southern Rocky Mountains represent a distinct transitional district [32] and the southern-most outpost for a number of arctic and boreal species.

Similar to *C. parryi*, we discovered that two samples of *C. scouleri* fall out in very different positions in the cpDNA phylogeny. An individual of unknown provenance and apparently lacking a voucher [3] lies within *Rapunculus* 2, while our own sample taken from the Olympic Peninsula in Washington State is placed in the Cordilleran clade (see Table S1 for Genbank and voucher numbers where available). We have confirmed the identity of our *C. scouleri* specimen and the voucher is archived in the Western Washington University Herbarium (WWB). Assuming that both samples were identified correctly, this raises the possibility that they represent morphologically similar but distinct and deeply divergent species. More comprehensive geographic sampling and molecular phylogenetic analyses will be necessary to evaluate this hypothesis.

In the cpDNA phylogeny, the occurrence of *C. divaricata* in the Cordilleran clade (subclade 1A) is notable because it represents the only non-northwestern member of the group. *Campanula divaricata* is a dry woodland species native to the eastern United States. However, its position among the northwestern species remains uncertain. In the ITS phylogeny the species falls within *Rapunculus* 2 instead. Several reasons may explain the incongruence between the nuclear and chloroplast markers. Both incomplete lineage sorting of ancestral polymorphisms and hybridization can produce disagreement among gene trees based on independent loci [34]. Hybridization is a common evolutionary force in plants [35], [36], [37] and has been documented in genera from all the major clades within Campanulaceae, including the *Campanula* s. str. [38] and *Rapunculus* clades [10], [30], [38]. Moreover, the opportunity for hybridization has existed for *C. divaricata*, as its range overlaps with other members of the *Rapunculus* 1 (*C. rotundifolia)* and *Rapunculus* 2 (*C. americana* and *Triodanis*) clades. Thus, while incomplete lineage sorting can not be ruled out as a possibility, based on other evidence within the Campanulaceae hybridization is a likely cause of the discrepancy between the nuclear and chloroplast trees. To more clearly determine the root of the incongruence, even if stochastic factors are the cause, sampling of eastern North American taxa and the use of additional nuclear markers are warranted.

In contrast to the hardy perennial habit and exclusively *Campanula* composition of *Rapunculus* 1, *Rapunculus* 2 is a collection of highly divergent genera that includes several Old World taxa and most North American Campanuloideae genera: *Triodanis*, *Githopsis* Nutt., *Heterocodon* Nutt., and annual species of *Campanula*. The North American taxa associated with this group are mostly narrow endemics belonging to monotypic genera or genera with limited species compositions, though they harbor much infraspecific variation [2], [22], [39]. Another distinction of the *Rapunculus 2* clade is the high incidence of plants with annual and biennial habits, perhaps in response to ephemeral moisture availability and arid environments.

The *Rapunculus* 2 clade shows three independent colonizations of North America by *Campanula* (Fig. 2). One of these events involved the ancestor of *C. uniflora*, which shows close ties to *Asyneuma*, *Phyteuma*, *Physoplexis* Schur, and *Petromarula* and represents a single colonization of northwestern North America (*Rapunculus* 2C; Fig. 2), consistent with the results of a previous molecular study based on *ITS* data alone [1]. *Campanula uniflora* exhibits a broad circum-Arctic distribution, penetrating deep into North America along the Rocky Mountains and inhabiting higher elevations at lower latitudes. The other two colonizations of North America were followed by episodes of diversification of endemic North American clades. One of these includes *C. americana* L. (previously known as the monotypic *Campanulastrum americanum* (L.) Small [23], [39] but see [2]), *C. reverchonii* A. Gray, and two species of *Triodanis*, *T. coloradoensis* (Buckley) McVaugh and *T. perfoliata* (L.) Nieuwl (*Rapunculus* 2B; Fig. 2). According to the ITS data, *C. divaricata* may also occur within this group. *Campanula reverchonii* occupies granitic outcroppings and was previously suggested as a relative of this group based on seed morphology [21]. *Campanula americana* is a robust biennial of moist habitats in the eastern United States. This clade is nested within the Mediterranean and European taxa of *Legousia*.

The other North American radiation in the *Rapunculus* 2 clade includes members of *Campanula*, *Githopsis*, and *Heterocodon* (*Rapunculus* 2A; Fig. 2). Shetler and Morin [21] suggested that these largely xeric-adapted annuals represent one of the campanuloid radiations in North America, and our data are consistent with this hypothesis. Diversification in this group appears to have centered on low latitudes relative to other North American *Campanula*. Several members of *Rapunculus* 2 are specifically associated with the California Floristic Province (e.g., *C. exigua* Rattan, *C. wilkinsiana* Greene, *C. prenanthoides*, and possibly *C. scouleri*), forming a narrowly distributed cluster of *Campanula* species that are phylogenetically and biogeographically distinct from species of the more northerly Rocky Mountain Floristic Province despite certain ecological similarities (e.g., *C. wilkinsiana* occupies high-elevation environments like the members of the Cordilleran clade).

Western North America is comprised of a geographically complex landscape with a high degree of environmental heterogeneity, affording plants a number of unique habitat opportunities. Accordingly, 60% of the 987 vascular plant genera endemic to North America are limited to the western portion of the continent [40]. The high proportion of geographically limited *Campanula* in western North America is likely a product of topographically driven isolation among neighboring populations. Indeed, specialized high elevation plants often inhabit an archipelago of ‘sky islands’ strung along the western cordillera [31]. Sky islands are not only separated by distance, but also by dispersal barriers such as the inhospitable environs of low elevation forests and sagelands in which cold-adapted species might not compete well for resources. Where the patchy distribution of suitable habitat along the alpine archipelago hampers gene flow, isolation has probably promoted genetic divergence among populations, and thus provided an opportunity for speciation. Longstanding isolation of sky island populations due to the natural fragmentation of high elevation habitats may explain the narrow geographic distributions observed for several closely related species inhabiting the western cordillera, though we recognize that species distributions may have fluctuated historically in response to Pleistocene climatic oscillations. In particular, cold-adapted alpine species might have thrived and undergone geographic expansion under glacial climate conditions. Patterns of population structure may therefore reflect a balance between glacial-age range expansion and interglacial range contraction [41].

The history of rapid radiation in the Campanulaceae [4] begs the question as to what ecological and evolutionary forces have promoted divergence in the family. Our phylogenetic analyses of Northwestern *Campanula* suggest that isolation of populations, potentially due to the heterogeneous topography of the region as well as its history of glaciations during the Pleistocene, promoted divergence leading to radiations and the generation of endemics in the Rocky Mountain Floristic Province. Though the family is not particularly speciose in North America, radiations have been documented for the lineages *Triodanis* and the *Campanula-Githopsis-Heterocodon* clade [22]. A high degree of endemism was also noted for Campanulaceae across the rugged landscape of Crete, though this is likely a result of the loss of mainland species rather than diversification [16]. Similar inferences of relictual endemics were made for Campanulaceae on oceanic islands, where, once established, diversification was limited [3]. This striking variation in the process and timing of speciation throughout the family and across the globe underscores the need for more complete sampling of populations within biogeographic regions, for the evolutionary history of a group depends on the demography, dispersal capacity and adaptability of the species involved, as well as the environmental heterogeneity of the local landscape.

Members of *Campanula* have colonized North America at least six times with subsequent radiations that gave rise to the diversity and high degree of endemism seen on the continent today. The vast majority of North American species are members of the *Rapanculus* clade, with *C. dasyantha* subsp. *chamissonis* being the only exception. Our molecular phylogenetic analyses also uncovered at least one morphologically cryptic species (*C. parryi* var. *parryi* versus *C. parryi* var. *idahoensis*) as well as compelling genetic differences in *C. scouleri* across its range. As such, these analyses have opened many doors for further inquiry, including i) a complete molecular analysis of North American *Campanula* with infraspecific taxa that would resolve questions about cryptic species and uncertainties in the phylogenetic placement of *C. scouleri* and *C. divaricata*, ii) a global molecular analysis of the *C. rotundifolia* complex to determine species-level delineations, and iii) an investigation into the phylogeography and timing of the radiations of North American *Campanula* to improve our understanding of speciation processes in this genus.

To resolve fine-scale relationships within the major clades identified in this study and elsewhere (e.g., [1], [3], [4]) it will be necessary to fill gaps in taxonomic sampling for the various genetic markers that have been the primary targets of phylogenetic studies to date. Expanding on available datasets for *atpB* and *matK* may be especially useful as these markers are undersampled, yet appear to have good resolving power across a range of phylogenetic scales.

## Materials and Methods

In order to infer the phylogenetic associations of northwestern *Campanula*, we incorporated genetic sequence data from multiple cpDNA markers for approximately 75% of the North American Campanuloideae [2], including 12 *Campanula* from the northwest, in a phylogenetic analysis of more than 100 campanuloid species worldwide. We performed further analyses to resolve the evolutionary relationships among members of the *Rapunculus* clade, in which all but one of the North American taxa occur, using additional cpDNA and nDNA markers.

### Taxonomic sampling and data collection

As a starting point for determining evolutionary relationships between North American taxa and other *Campanula* species, we obtained all Campanulaceae sequence data available from Genbank for five molecular markers that have been widely used by other investigators. These markers included the cpDNA genes *atpB*, *rbcL* and *matK*, the intergenic spacer between the cpDNA's trnL and trnF regions (*trnL-F*), and the first and second internal transcribed spacers of nDNA ribosomal DNA (ITS). Data obtained from Genbank were drawn from 16 published and 1 unpublished study, including large contributions by Haberle et al. [3] (*atpB*, *rbcL*, *matK*), Roquet et al. [1], [4] (*trnL-F*, ITS), and Eddie et al. [14] (ITS). The full list of Genbank numbers for sequences used in the study is given in Table S1.

In addition to the previously published data, we collected new DNA sequence data from 11 North American taxa (*C. dasyantha* subsp. *chamissonis*, *C. uniflora*, *C. rotundifolia*, *C. lasiocarpa*, *C. aurita*, *C. piperi*, *C. scabrella*, *C. parryi* var. *idahoensis*, *C. parryi* var. *parryi*, *C. scouleri*, and *C. wilkinsiana* and one European species (*C. cochlearifolia* Lam.). Though our emphasis was on North American taxa, we collected new data from *C. cochlearifolia* because preliminary analyses based on limited data available from Genbank suggested that it may be closely related to certain North American species. For most taxa we sampled at least two individuals representing different populations. Given *C. rotundifolia's* wide distribution and the possibility that it represents a species complex [2], we sequenced five individuals from across its range, 3 from North America and 2 from Europe, to add to data from previous studies [3].

We obtained *Campanula* specimens for DNA sequencing either directly from the field or via herbarium loans (see Table S1 for herbarium accession numbers). Freshly collected wild specimens were stored on ice or packed in silica crystals prior to storage at −20°C. Representative voucher specimens, collected in conjunction with all specimens intended for DNA extraction, were archived at the Western Washington University Herbarium (WWB). Total genomic DNA was isolated from leaf, stem or flower tissue using the DNeasy Plant Mini Kit (Qiagen, Valencia, California). The androecium and gynoecium were avoided to reduce the potential of incorporating recombinant DNA from ovules and pollen.

All molecular markers were PCR amplified under the following conditions: 4 min (94°C); 35 cycles of 1 min denaturing (94°C), 1 min annealing (atpB, rbcL, matK: 58°C; *trnL-F*: 53°C; ITS: 48°C), and 1 min extension (72°C); and a final extension of 10 min (72°C). Primers for *atpB* (S2F, S1494R, S611F), *rbcL* (1F, CNEWR, C427MF) and *matK* (5F, C745R, C745F, C2MR) are described in Haberle et al. [3]. For *trnL-F* we used primers trnLb2(UAA) and trnF(GAA) [42]. ITS was amplified using primers 17SE and 26SE [43]. ITS and *trnL-F* PCR products were cloned using TOPO TA cloning kits (Invitrogen, Carlsbad, California) prior to sequencing. All other markers were sequenced directly from PCR products. Purified plasmids from clones or PCR products were sequenced in both directions by Nevada Genomics (Reno, NV) or the University of Washington's High Throughput Genomics Unit (Seattle, WA). Sequences were manually edited in Sequencher (Gene Codes Corp.) and archived in GenBank (*atpB*: JN571924-JN571942; *rbcL*: JN571965-JN571987; *matK*: JN571943-JN571964; *trnL-F*: JN571998-JN572026; ITS: JN571988-JN571997).

### Phylogenetic Analyses

All sequence datasets were aligned using ClustalW as implemented in MEGA v4 [44] and alignments were checked by eye. Efforts to align *trnL-F* and ITS data for all available Campanulaceae revealed several regions of ambiguous homology and numerous indels due to high levels of variation among the many deeply divergent evolutionary lineages within the family. Because of the uncertainty in the accuracy of high-level alignments, we limited the *trnL-F* and ITS datasets to only include members of the *Rapunculus* clade of Campanulaceae [4], which preliminary analyses showed to be the clade that includes the majority of our focal taxa. The lower levels of genetic variation evident within the *Rapunculus* group relative to the complete family allowed more robust alignments. As outgroups for the family-wide analyses we selected *Cyphia elata* Harv., *Pseudonemacladus oppositifolius* (B.L. Rob.) McVaugh, *Solenopsis minuta* C. Presl, *S. laurentia* C. Presl, and *Lobelia cardinalis* L. after Haberle et al. [3]. *Campanula latifolia* L. and *C. erinus* L. of the *Campanula* s. str. clade [4], which is sister to the *Rapunculus* clade, were chosen as outgroups for the taxonomically focused analyses.

To minimize the potentially confounding effect of reticulate evolution on phylogenetic reconstruction, we tested the datasets representing each genetic marker for recombination using the RDP [45], MAXCHI and CHIMAERA [46], and GENECOV [47] algorithms implemented in the software RDP3 [48]. Sequences that showed similar recombinant signatures under two or more of these methods were discarded. We removed putatively recombinant sequences from the *matK* (Genbank # EU713328, EU713263) and *rbcL* (EU713353) datasets. Species represented by these sequences (*Trachelium caerulium* L., *L. cardinalis*, *Canarina canariensis* Kuntze) were also excluded from concatenated analyses (see below). Except as described below, we excluded indels and sites of ambiguous alignment from further analyses.

We conducted seven distinct sets of phylogenetic analyses. First, we analyzed all available data for each of the five molecular markers in separate analyses. This allowed us to evaluate relationships among all individuals and species for which data are available, including those that are represented by only a single marker. Second, we analyzed a family-wide dataset (one representative individual per species) consisting of concatenated *atpB*, *rbcL*, and *matK* sequences. This analysis allowed us to place our focal taxa within the broader context of the Campanulaceae with better phylogenetic resolution than was possible under the single-marker analyses. Finally, we performed a concatenated analysis specifically focused on the *Rapunculus* clade [4] based on *atpB*, *rbcL*, *matK*, and *trnL-F*. We did not include ITS in this concatenated analysis because it is unlinked to the cpDNA markers and therefore may have a different underlying evolutionary history. Where data were available, multiple individuals of the focal taxa were included in this analysis, offering a limited test of monophyly for those species. In both concatenated analyses, species or individuals were included only if they were represented by data from at least two molecular markers. As much as possible, concatenated sequences were drawn from a single individual; however, in some cases data from different individuals of the same species were combined to maximize the number of species that could be included in the analyses [49].

We used ML, Bayesian, and MP methods to reconstruct phylogenies. We first selected models of nucleotide substitution for each molecular marker using DT-ModSel [50] based on ML starting trees generated using GARLI v0.96 [51] under the general time reversible + proportion of invariant sites (I) + gamma (Γ) model. Selected models were as follows: *atpB* – variable transversion rate model (TVM)+I+Γ; *rbcL*−TVM+I+Γ; *matK*−TVM+Γ; *trnL-F*−TVM+Γ; ITS – symmetrical model (SYM)+Γ [52] . Optimal ML trees for individual markers were identified using GARLI under appropriate substitution models (ten replicates per analysis). For ML analyses on concatenated datasets we used GARLI-PART v0.97 [53] (10 replicates per analysis), which allowed concatenated data to be partitioned by marker with appropriate substitution models applied to each partition. For all ML analyses we evaluated nodal support using 100 bootstrap replicates in the appropriate version of GARLI (2 analysis replicates per bootstrap).

Bayesian phylogenetic analyses were performed using MRBAYES v3.1.2 [54]. Models of nucleotide evolution and partitioning schemes were applied as described above. Rates of evolution were allowed to vary among partitions in the concatenated analysis focused on *Rapunculus*. For the family-wide concatenated analysis, we applied a fixed rate due to difficulties reaching stationarity under the variable rate model. With the exception of the single-marker *rbcL* analysis (discussed below), run length varied between 10 and 20 million generations for different datasets depending on the time it took to achieve stationarity. All analyses included 5 chains and 5000 samples drawn from the posterior, and each was repeated at least 3 times from different random seeds to confirm convergence on similar topologies. Stationarity and burn-in length were assessed by visualizing posterior distributions in Tracer v1.5 [55], ensuring that the standard deviation of split frequencies approached zero (all <0.01), and confirming that independent runs converged on similar tree topologies using AWTY [56]. A 10% burn-in was sufficient for all analyses.

The Bayesian analysis based on *rbcL* alone showed persistent failure to converge on a single topology. We conducted 8 separate analyses ranging from 20 to 30 million generations in length and otherwise set up as described above. Inspection of the resulting tree topologies showed that most strongly supported relationships were consistently retrieved in all analyses. To evaluate the possibility that different rates of evolution at different codon positions may have contributed to the ambiguous result for this locus, we ran an additional Bayesian analysis in which the marker was partitioned by codon position and a separate substitution model (selected as described above) was applied for each partition. This did not produce a substantively different tree from the original analysis, nor did it appear to correct the problem of convergence. We therefore present the results of the original runs, treating relationships that differed between trees as having low support. Topological ambiguity was often associated with a specific sequence representing *C. rapunculoides* L. (Genbank # FJ587271) (Fig. S3).

Finally, we used PAUP* [57] to evaluate robustness of tree topologies under a MP framework. For each dataset we performed 500 bootstrap replicates with heuristic searches, TBR branch swapping, 10 random addition replicates, and maxtrees limited to 100. Though we excluded indels from all prior analyses, we found that the ITS dataset included several potentially parsimony-informative indels. Therefore, we conducted additional MP bootstrap analyses on the ITS dataset, applying both SIC (each indel coded as a binary character; [58]) and MCIC (indels coded as multi-state characters; [59]). Indels were coded using SeqState v1.4.1 [60].

## Supporting Information

Table S1 Species and sequence data used for phylogenetic analysis of the Campanulaceae. For each species we list the major clade in which it falls, herbarium voucher number (only for newly sequenced species/individuals from this study), Genbank numbers for available sequence data, and citations for previously published data (see reference list following table). Species names marked with an asterisk (*) have been updated from the names originally listed on the Genbank accessions to be consistent with the classification scheme of Lammers (2007). Similarly, *Asyneuma comosiforme* has been changed to *Campanula comosiformis* based on the findings of Frajman and Schneeweiss (2009). A cross (†) next to a species name indicates ambiguity in the placement of the species within major clades due to incongruent results from different individuals or genetic markers. Uncertainty in clade assignments due to poor phylogenetic resolution is denoted by a question mark (?) in the “Clade” column.
(DOC)

Figure S1 **Best maximum likelihood phylogeny for the Campanulaceae based on all available** ***atpB*** **DNA sequences.** Outgroups have been removed for clarity. Numbers after species names are Genbank accession numbers for sequences used to build the tree. Pie graphs on branches indicate relationships that are well supported under one or more of the three tree-building methods that were employed. Black-filled segments of the graphs indicate maximum parsimony (upper left) or maximum likelihood (upper right) bootstrap values >75, or Bayesian posterior probabilities >0.95 (bottom).
(EPS)

Figure S2 **Best maximum likelihood phylogeny for the Campanulaceae based on all available** ***matK*** **DNA sequences.** Outgroups have been removed for clarity. Numbers after species names are Genbank accession numbers for sequences used to build the tree. Pie graphs on branches indicate relationships that are well supported under one or more of the three tree-building methods that were employed. Black-filled segments of the graphs indicate maximum parsimony (upper left) or maximum likelihood (upper right) bootstrap values >75, or Bayesian posterior probabilities >0.95 (bottom).
(EPS)

Figure S3 **Best maximum likelihood phylogeny for the Campanulaceae based on all available** ***rbcL*** **DNA sequences.** Outgroups have been removed for clarity. Numbers after species names are Genbank accession numbers for sequences used to build the tree. Pie graphs on branches indicate relationships that are well supported under one or more of the three tree-building methods that were employed. Black-filled segments of the graphs indicate maximum parsimony (upper left) or maximum likelihood (upper right) bootstrap values >75, or Bayesian posterior probabilities >0.95 (bottom). Black arrow marks *Campanula rapunculoides* (Genbank # FJ587271), which some Bayesian analyses placed in an alternative position in the tree (grey text and arrow). The topology shown here is consistent with the most commonly retrieved Bayesian tree.
(EPS)

Figure S4 **Best maximum likelihood phylogeny for the** ***Rapunculus*** **clade based on all available** ***trnL-F*** **DNA sequences.** Outgroups have been removed for clarity. Numbers after species names are Genbank accession numbers for sequences used to build the tree. Pie graphs on branches indicate relationships that are well supported under one or more of the three tree-building methods that were employed. Black-filled segments of the graphs indicate maximum parsimony (upper left) or maximum likelihood (upper right) bootstrap values >75, or Bayesian posterior probabilities >0.95 (bottom).
(EPS)
