# Supplementary material for: Resolving the Evolutionary History of Campanula (Campanulaceae) in Western North America
Source: PLoS One. 2011 Sep 9;6(9):e23559. doi: 10.1371/journal.pone.0023559 (PMC3170292; doi:10.1371/journal.pone.0023559)
Supplement: Table S1 — Species and sequence data used for phylogenetic analysis of the Campanulaceae. For each species we list the major clade in which it falls, herbarium voucher number (only for newly sequenced species/individuals from this study), Genbank numbers for available sequence data, and citations for previously published data (see reference list following table). Species names marked with an asterisk (*) have been updated from the names originally listed on the Genbank accessions to be consistent with the classification scheme of Lammers (2007). Similarly, Asyneuma comosiforme has been changed to Campanula comosiformis based on the findings of Frajman and Schneeweiss (2009). A cross (†) next to a species name indicates ambiguity in the placement of the species within major clades due to incongruent results from different individuals or genetic markers. Uncertainty in clade assignments due to poor phylogenetic resolution is denoted by a question mark (?) in the “Clade” column. (DOC) [file pone.0023559.s001.doc]

**Table S1.** Species and sequence data used for phylogenetic analysis of the Campanulaceae. For each species we list the major clade in which it falls, herbarium voucher number (only for newly sequenced species/individuals from this study), Genbank numbers for available sequence data, and citations for previously published data (see reference list following table). Species names marked with an asterisk (*) have been updated from the names originally listed on the Genbank accessions to be consistent with the classification scheme of Lammers (2007). Similarly, *Asyneuma comosiforme* has been changed to *Campanula comosiformis* based on the findings of Frajman and Schneeweiss (2009). A cross (†) next to a species name indicates ambiguity in the placement of the species within major clades due to incongruent results from different individuals or genetic markers. Uncertainty in clade assignments due to poor phylogenetic resolution is denoted by a question mark (?) in the "Clade" column.

|  |  |  | **atpB** | **matK** | **rbcL** | **trnL-F** | **ITS** |  |
| --- | --- | --- | --- | --- | --- | --- | --- | --- |
| **Species** | **Clade** | **Voucher** | **Genbank** | **Genbank** | **Genbank** | **Genbank** | **Genbank** | **Source** |
| *Adenophora divaricata* | *Rapunculus* 1 |  |  |  |  |  | AF090710, AF090711 | 7 |
| *Adenophora divaricata* | *Rapunculus* 1 |  | EU437656 | EU713323 | EU713430 |  |  | 8 |
| *Adenophora himalayana* | *Rapunculus* 1 |  |  |  |  |  | AF090716, AF090717 | 7 |
| *Adenophora lobophylla* | *Rapunculus* 1 |  |  |  |  |  | AF090706, AF090707 | 7 |
| *Adenophora morrisonensis* | *Rapunculus* 1 |  |  |  |  |  | AF090718, AF090719 | 7 |
| *Adenophora paniculata* | *Rapunculus* 1 |  |  |  |  |  | AF090714, AF090715 | 7 |
| *Adenophora petiolata* | *Rapunculus* 1 |  |  |  |  |  | AF090700, AF090701 | 7 |
| *Adenophora potaninii* | *Rapunculus* 1 |  |  |  |  |  | AF090704, AF090705 | 7 |
| *Adenophora remotiflora* | *Rapunculus* 1 |  |  |  |  |  | AY322006, AY331419 | 5 |
| *Adenophora remotiflora* | *Rapunculus* 1? |  |  |  |  | EF088693 |  | 12 |
| *Adenophora remotiflora* | *Rapunculus* 1 |  |  |  | EU643715 |  |  | 13 |
| *Adenophora remotiflora* | *Rapunculus* 1 |  |  |  |  |  | AY548197 | unpubl |
| *Adenophora stenanthina* | *Rapunculus* 1 |  |  |  |  |  | AF090708, AF090709 | 7 |
| *Adenophora stricta* | *Rapunculus* 1 |  |  |  |  |  | AF090712, AF090713 | 7 |
| *Adenophora stricta** | *Rapunculus* 1 |  |  |  | AY655145 |  |  | 4 |
| *Adenophora triphylla* | *Rapunculus* 1 |  |  |  |  |  | AY548194 | unpubl |
| *Adenophora triphylla* | *Rapunculus* 1 |  |  |  |  |  | AY548193 | unpubl |
| *Adenophora wawreana* | *Rapunculus* 1 |  |  |  |  |  | AF090702, AF090703 | 7 |
| *Asyneuma anthericoides* | *Rapunculus* 2 |  |  |  |  | GQ254891 |  | 15 |
| *Asyneuma campanuloides* | *Rapunculus* 2 |  |  |  |  | FJ426570 |  | 6 |
| *Asyneuma campanuloides* | *Rapunculus* 2 |  |  |  |  |  | DQ304586 | 11 |
| *Asyneuma canescens* | *Rapunculus* 2 |  |  |  |  | GQ254898 |  | 15 |
| *Asyneuma canescens* | *Rapunculus* 2 |  |  |  |  | GQ254899 |  | 15 |
| *Asyneuma japonicum* | *Rapunculus* 2 |  |  |  |  |  | AF183437, AF183438 | 9 |
| *Asyneuma limoniifolium* | *Rapunculus* 2 |  |  |  |  | FJ426571 |  | 6 |
| *Asyneuma limoniifolium* | *Rapunculus* 2 |  |  |  |  | EF088694 | EF090520, EF090561 | 12 |
| *Asyneuma limoniifolium* | *Rapunculus* 2 |  |  |  | EU643704 |  |  | 13 |
| *Asyneuma lobelioides* | *Rapunculus* 2 |  |  |  |  | FJ426568 |  | 6 |
| *Asyneuma lobelioides* | *Rapunculus* 2 |  |  |  |  | EF088695 |  | 12 |
| *Asyneuma lobelioides* | *Rapunculus* 2 |  |  |  | EU643733 |  |  | 13 |
|  |  |  | **atpB** | **matK** | **rbcL** | **trnL-F** | **ITS** |  |
| **Species** | **Clade** | **Voucher** | **Genbank** | **Genbank** | **Genbank** | **Genbank** | **Genbank** | **Source** |
| *Asyneuma pichleri* | *Rapunculus* 2 |  |  |  |  | FJ426569 |  | 6 |
| *Asyneuma pichleri* | *Rapunculus* 2 |  |  |  |  | GQ254911 |  | 15 |
| *Asyneuma pichleri* | *Rapunculus* 2 |  |  |  |  | GQ254913 |  | 15 |
| *Asyneuma pichleri* | *Rapunculus* 2 |  |  |  |  | GQ254914 |  | 15 |
| *Asyneuma trichocalycinum* | *Rapunculus* 2 |  |  |  |  | FJ426566 |  | 6 |
| *Asyneuma virgatum* | *Rapunculus* 2 |  |  |  | AY655146 |  |  | 4 |
| *Asyneuma virgatum* | *Rapunculus* 2 |  | EU437665 | EU713332 | EU713439 |  |  | 8 |
| *Azorina vidalii* | *Campanula* s. str. |  | EU437601 | EU713266 | EU713373 |  |  | 8 |
| *Azorina vidalii* | *Campanula* s. str. |  |  |  | EU643713 |  |  | 13 |
| *Berenice arguta* | Wahlenbergieae |  | EU437672 | EU713339 | EU713446 |  |  | 8 |
| *Campanula aizoides* | *Rapunculus* 1 |  | EU437662 | EU713329 | EU713436 |  |  | 8 |
| *Campanula alata** | *Musschia* clade |  |  |  | EU643718 |  |  | 13 |
| *Campanula alliariifolia* | *Campanula* s. str. |  | EU437604 | EU713269 | EU713376 |  |  | 8 |
| *Campanula alliariifolia* | *Campanula* s. str. |  |  |  | FJ587241 |  |  | 13 |
| *Campanula americana** | *Rapunculus* 2 |  |  |  |  |  | AY322044, AY331457 | 5 |
| *Campanula americana** | *Rapunculus* 2 |  | EU437646 | EU713312 | EU713419 |  |  | 8 |
| *Campanula americana** | *Rapunculus* 2 |  |  |  |  | EF088776 |  | 12 |
| *Campanula americana** | *Rapunculus* 2 |  |  |  | EU643729 |  |  | 13 |
| *Campanula americana** | *Rapunculus* 2 |  |  |  |  | GQ254928 |  | 15 |
| *Campanula aparinoides* | *Rapunculus* 2 |  |  |  |  | EF088702 |  | 12 |
| *Campanula aparinoides* | *Rapunculus* 2 |  |  |  | EU643728 |  |  | 13 |
| *Campanula armena** | *Campanula* s. str. |  | EU437611 | EU713276 | EU713383 |  |  | 8 |
| *Campanula armena** | *Campanula* s. str. |  |  |  | FJ587242 |  |  | 13 |
| *Campanula arvatica* | *Rapunculus* 1 |  |  |  |  |  | AY322010, AY331423 | 5 |
| *Campanula arvatica* | *Rapunculus* 1 |  | EU437677 | EU713344 | EU713451 |  |  | 8 |
| *Campanula asperuloides* | *Trachelium* clade |  |  |  | DQ356117 |  |  | 1 |
| *Campanula aurita* | *Rapunculus* 1 | AKA 17395 |  | JN571943 | JN571966 | JN571998 | JN571991 |  |
| *Campanula aurita* | *Rapunculus* 1 | AKA V122137 | JN571924 | JN571944 | JN571965 | JN571999 |  |  |
| *Campanula aurita* | *Rapunculus* 1 | AKA 89904 |  |  |  | JN572000 |  |  |
| *Campanula balfourii* | *Campanula* s. str. |  |  |  | FJ587243 |  |  | 13 |
| *Campanula baumgartenii** | *Rapunculus* 1 |  |  |  |  |  | DQ304619 | 11 |
| *Campanula bellidifolia* | *Campanula* s. str. |  | EU437575 | EU713240 | EU713347 |  |  | 8 |
| *Campanula bellidifolia* | *Campanula* s. str. |  |  |  | FJ587244 |  |  | 13 |
| *Campanula bellidifolia** | *Campanula* s. str. |  | EU437576 | EU713241 | EU713348 |  |  | 8 |
| *Campanula bellidifolia** | *Campanula* s. str. |  |  |  | FJ587274 |  |  | 13 |
| *Campanula betulifolia* | *Campanula* s. str. |  |  |  | FJ587245 |  |  | 13 |
| *Campanula bononiensis* | *Campanula* s. str. |  | EU437609 | EU713274 | EU713381 |  |  | 8 |
| *Campanula cabezudoi* | *Rapunculus* 1 |  |  |  |  |  | FM212727 | 3 |
| *Campanula cabezudoi* | *Rapunculus* 1 |  |  |  |  |  | FM212728 | 3 |
| *Campanula cabezudoi* | *Rapunculus* 1 |  |  |  |  |  | FM212729 | 3 |
|  |  |  | **atpB** | **matK** | **rbcL** | **trnL-F** | **ITS** |  |
| **Species** | **Clade** | **Voucher** | **Genbank** | **Genbank** | **Genbank** | **Genbank** | **Genbank** | **Source** |
| *Campanula cabezudoi* | *Rapunculus* 1 |  |  |  |  |  | FM212730 | 3 |
| *Campanula cabezudoi* | *Rapunculus* 1 |  |  |  |  |  | FM212731 | 3 |
| *Campanula cabezudoi* | *Rapunculus* 1 |  |  |  |  |  | FM212732 | 3 |
| *Campanula carpatha* | *Campanula* s. str. |  | EU437596 | EU713261 | EU713368 |  |  | 8 |
| *Campanula carpatica* | *Rapunculus* 1 |  |  |  |  |  | AY322013, AY331426 | 5 |
| *Campanula carpatica* | *Rapunculus* 1 |  |  | EU713303 | EU713410 |  |  | 8 |
| *Campanula cenisia* | *Rapunculus* 1 |  |  |  |  |  | DQ304622 | 11 |
| *Campanula cenisia* | *Rapunculus* 1 |  |  |  |  |  | DQ304623 | 11 |
| *Campanula cespitosa* | *Rapunculus* 1 |  |  |  |  |  | DQ304621 | 11 |
| *Campanula cochleariifolia* | *Rapunculus* 1 |  |  |  |  | EF088710 |  | 12 |
| *Campanula cochleariifolia* | *Rapunculus* 1 |  |  |  | FJ587247 |  |  | 13 |
| *Campanula cochleariifolia* | *Rapunculus* 1 | RBGE 2037 | JN571926 | JN571946 |  |  |  |  |
| *Campanula collina* | *Campanula* s. str. |  |  |  | FJ587248 |  |  | 13 |
| *Campanula comosiformis* | *Rapunculus* 1 |  |  |  |  | FJ426572 | FJ426592 | 6 |
| *Campanula conferta* | *Campanula* s. str. |  |  |  | FJ587249 |  |  | 13 |
| *Campanula cretica* | *Rapunculus* 2 |  | EU437663 | EU713330 | EU713437 |  |  | 8 |
| *Campanula creutzburgii* | *Campanula* s. str. |  | EU437625 | EU713290 | EU713397 |  |  | 8 |
| *Campanula creutzburgii* | *Campanula* s. str. |  |  |  | EU643730 |  |  | 13 |
| *Campanula cymbalaria* | *Rapunculus* 2 ? |  |  |  |  | EF088715 |  | 12 |
| *Campanula dasyantha chamissonis** | *Campanula* s. str. |  |  |  | EU643724 |  |  | 13 |
| *Campanula dasyantha chamissonis** | *Campanula* s. str. | ALA V149556 | JN571925 | JN571945 | JN571967 | JN572023 | JN571996 |  |
| *Campanula dasyantha chamissonis** | *Campanula* s. str. | ALA V74534 |  |  |  | JN572024 |  |  |
| *Campanula dasyantha chamissonis** | *Campanula* s. str. | ALA V91312 |  |  |  | JN572025 |  |  |
| *Campanula decumbens* | *Rapunculus* 1 |  |  |  |  | EF088716 | EF090526, EF090567 | 12 |
| *Campanula decumbens* | *Rapunculus* 1 |  |  |  | FJ587250 |  |  | 13 |
| *Campanula decumbens** | *Rapunculus* 1 |  |  |  |  |  | FM212733 | 3 |
| *Campanula decumbens** | *Rapunculus* 1 |  |  |  |  |  | FM212734 | 3 |
| *Campanula decumbens** | *Rapunculus* 1 |  |  |  |  |  | FM212735 | 3 |
| *Campanula dichotoma* | *Campanula* s. str. |  |  |  | FJ587251 |  |  | 13 |
| *Campanula dimorphantha* | *Campanula* s. str. |  |  |  | FJ587246 |  |  | 13 |
| *Campanula divaricata*† | *Rapunculus* 2 |  |  |  |  |  | AY322014, AY331427 | 5 |
| *Campanula divaricata*† | *Rapunculus* 1 |  | EU437676 | EU713343 | EU713450 |  |  | 8 |
| *Campanula divaricata*† | *Rapunculus* 1 |  |  |  |  | EF088718 |  | 12 |
| *Campanula divaricata*† | *Rapunculus* 1 |  |  |  | EU643732 |  |  | 13 |
| *Campanula drabifolia* | *Campanula* s. str. |  |  |  | FJ587252 |  |  | 13 |
| *Campanula edulis* | *Campanula* s. str. |  | EU437602 | EU713267 | EU713374 |  |  | 8 |
| *Campanula elatines* | *Rapunculus* 1 |  |  |  | AY655147 |  |  | 4 |
| *Campanula elatines* | *Rapunculus* 1 |  |  |  |  | FJ426577 |  | 6 |
| *Campanula elatines* | *Rapunculus* 1 |  | EU437664 | EU713331 | EU713438 |  |  | 8 |
| *Campanula elatines* | *Rapunculus* 1 |  |  |  |  |  | DQ304624 | 11 |
|  |  |  | **atpB** | **matK** | **rbcL** | **trnL-F** | **ITS** |  |
| **Species** | **Clade** | **Voucher** | **Genbank** | **Genbank** | **Genbank** | **Genbank** | **Genbank** | **Source** |
| *Campanula elatinoides* | *Rapunculus* 1 |  |  |  |  | FJ426578 |  | 6 |
| *Campanula elatinoides* | *Rapunculus* 1 |  |  |  |  |  | DQ304625 | 11 |
| *Campanula erinus* | *Campanula* s. str. |  | EU437626 | EU713291 | EU713398 |  |  | 8 |
| *Campanula erinus* | *Campanula* s. str. |  |  |  |  |  | DQ304580 | 11 |
| *Campanula erinus* | *Campanula* s. str. |  |  |  |  | EF088720 |  | 12 |
| *Campanula erinus* | *Campanula* s. str. |  |  |  | EU643734 |  |  | 13 |
| *Campanula exigua* | *Rapunculus* 2 |  | EU437643 | EU713309 | EU713416 |  |  | 8 |
| *Campanula fastigiata* | *Rapunculus* 1 ? |  |  |  |  | EF088721 | EF090529, EF090570 | 12 |
| *Campanula fastigiata* | *Rapunculus* 1 ? |  |  |  | EU643727 |  |  | 13 |
| *Campanula fenestrellata* | *Rapunculus* 1 |  |  |  |  | AJ430970 |  | 2 |
| *Campanula fenestrellata* | *Rapunculus* 1 |  |  |  |  | FJ426579 |  | 6 |
| *Campanula fenestrellata* | *Rapunculus* 1 |  |  |  |  | FJ426584 |  | 6 |
| *Campanula fenestrellata* | *Rapunculus* 1 |  |  |  |  |  | DQ304592 | 11 |
| *Campanula fenestrellata** | *Rapunculus* 1 |  |  |  |  | FJ426575 | DQ304595 | 11 |
| *Campanula filicaulis* | *Rapunculus* 1 |  |  |  | FJ587253 |  |  | 13 |
| *Campanula foliosa* | *Campanula* s. str. |  |  |  | FJ587254 |  |  | 13 |
| *Campanula fragilis* | *Rapunculus* 1 |  |  |  |  | FJ426580 |  | 6 |
| *Campanula fragilis* | *Rapunculus* 1 |  | EU437655 | EU713321 | EU713428 |  |  | 8 |
| *Campanula fragilis* | *Rapunculus* 1 |  |  |  |  |  | DQ304629 | 11 |
| *Campanula fragilis* | *Rapunculus* 1 |  |  |  |  |  | DQ304626 | 11 |
| *Campanula fragilis* | *Rapunculus* 1 |  |  |  |  |  | DQ304627 | 11 |
| *Campanula fragilis* | *Rapunculus* 1 |  |  |  |  |  | DQ304628 | 11 |
| *Campanula fruticulosa* | *Trachelium* clade |  |  |  | EU643716 |  |  | 13 |
| *Campanula garganica* | *Rapunculus* 1 |  |  |  |  | FJ426581 |  | 6 |
| *Campanula garganica* | *Rapunculus* 1 |  |  |  |  |  | DQ304596 | 11 |
| *Campanula garganica* | *Rapunculus* 1 |  |  |  |  | EF088725 | EF090532, EF090573 | 12 |
| *Campanula garganica* | *Rapunculus* 1 |  |  |  | FJ587255 |  |  | 13 |
| *Campanula garganica* | *Rapunculus* 1 |  |  |  |  | EF213145 |  | 16 |
| *Campanula garganica** | *Rapunculus* 1 |  |  |  |  | FJ426576 | DQ304597 | 11 |
| *Campanula garganica** | *Rapunculus* 1 |  |  |  |  |  | DQ304598 | 11 |
| *Campanula glomerata** | *Campanula* s. str. |  |  |  | FJ587284 |  |  | 13 |
| *Campanula haradjanii* | *Rapunculus* 1 |  |  |  |  | EF088726 |  | 12 |
| *Campanula hawkinsiana* | *Rapunculus* 1 |  |  |  |  |  | AY322019, AY331432 | 5 |
| *Campanula hawkinsiana* | *Rapunculus* 1 |  | EU437671 | EU713338 | EU713445 |  |  | 8 |
| *Campanula hawkinsiana* | *Rapunculus* 1 |  |  |  |  | EF213146 |  | 16 |
| *Campanula hercegovina* | *Rapunculus* 1 |  |  |  |  |  | DQ304616 | 11 |
| *Campanula hercegovina* | *Rapunculus* 1 |  |  |  |  |  | DQ304617 | 11 |
| *Campanula hercegovina* | *Rapunculus* 1 |  |  |  |  |  | DQ304618 | 11 |
| *Campanula herminii* | *Rapunculus* 1 |  |  |  |  |  | AY322020, AY331432 | 5 |
| *Campanula herminii* | *Rapunculus* 1 |  | EU437673 | EU713340 | EU713447 |  |  | 8 |
|  |  |  | **atpB** | **matK** | **rbcL** | **trnL-F** | **ITS** |  |
| **Species** | **Clade** | **Voucher** | **Genbank** | **Genbank** | **Genbank** | **Genbank** | **Genbank** | **Source** |
| *Campanula hierapetrae* | *Campanula* s. str. |  | EU437623 | EU713288 | EU713395 |  |  | 8 |
| *Campanula hofmannii** | *Campanula* s. str. |  |  |  | AY655159 |  |  | 4 |
| *Campanula hofmannii** | *Campanula* s. str. |  | EU437605 | EU713270 | EU713377 |  |  | 8 |
| *Campanula incurva* | *Campanula* s. str. |  |  |  | FJ587256 |  |  | 13 |
| *Campanula involucrata* | *Campanula* s. str. |  |  |  | FJ587257 |  |  | 13 |
| *Campanula isophylla* | *Rapunculus* 1 |  |  |  |  | FJ426583 |  | 6 |
| *Campanula isophylla* | *Rapunculus* 1 |  |  |  |  |  | DQ304630 | 11 |
| *Campanula jacquinii* | *Campanula* s. str. |  | EU437674 | EU713341 | EU713448 |  |  | 8 |
| *Campanula justiniana* | *Rapunculus* 1 |  |  |  |  |  | DQ304613 | 11 |
| *Campanula laciniata* | *Campanula* s. str. |  | EU437579 | EU713244 | EU713351 |  |  | 8 |
| *Campanula lactiflora** | *Musschia* clade |  | EU437652 | EU713318 | EU713425 |  |  | 8 |
| *Campanula lactiflora** | *Musschia* clade |  |  |  | EU643703 |  |  | 13 |
| *Campanula lanata* | *Campanula* s. str. |  | EU437610 | EU713275 | EU713382 |  |  | 8 |
| *Campanula lanata* | *Campanula* s. str. |  |  |  | FJ587259 |  |  | 13 |
| *Campanula lasiocarpa* | *Rapunculus* 1 |  |  |  |  | AB219602 |  | 14 |
| *Campanula lasiocarpa* | *Rapunculus* 1 | AKA V084236 | JN571928 | JN571947 | JN571968 | JN572001 |  |  |
| *Campanula lasiocarpa* | *Rapunculus* 1 | AKA 19612 |  |  |  | JN572002 |  |  |
| *Campanula lasiocarpa* | *Rapunculus* 1 | AKA V157726 |  |  |  | JN572003 |  |  |
| *Campanula lasiocarpa* | *Rapunculus* 1 | WWB 22731 |  |  |  | JN572004 |  |  |
| *Campanula lasiocarpa* | *Rapunculus* 1 | WWB 22732 | JN571927 | JN571948 | JN571969 | JN572005 | JN571992 |  |
| *Campanula lasiocarpa* | *Rapunculus* 1 | WWB 22741 |  |  |  | JN572006 |  |  |
| *Campanula lasiocarpa* | *Rapunculus* 1 | WS 355788 |  |  |  | JN572007 |  |  |
| *Campanula latifolia* | *Campanula* s. str. |  |  |  |  |  | AY322024, AY331437 | 5 |
| *Campanula latifolia* | *Campanula* s. str. |  | EU437606 | EU713271 | EU713378 |  |  | 8 |
| *Campanula latifolia* | *Campanula* s. str. |  |  |  |  | EF088732 |  | 12 |
| *Campanula latifolia* | *Campanula* s. str. |  |  |  | FJ587260 |  |  | 13 |
| *Campanula lusitanica* | *Rapunculus* 1 |  |  |  |  |  | FM212701 | 3 |
| *Campanula lusitanica* | *Rapunculus* 1 |  |  |  |  |  | FM212702 | 3 |
| *Campanula lusitanica* | *Rapunculus* 1 |  |  |  |  |  | FM212703 | 3 |
| *Campanula lusitanica* | *Rapunculus* 1 |  |  |  |  |  | FM212704 | 3 |
| *Campanula lusitanica* | *Rapunculus* 1 |  |  |  |  |  | AY322025, AY331438 | 5 |
| *Campanula lusitanica* | *Rapunculus* 1 |  | EU437667 | EU713334 | EU713441 |  |  | 8 |
| *Campanula lusitanica* | *Rapunculus* 1 |  |  |  |  | EF088733 |  | 12 |
| *Campanula lusitanica* | *Rapunculus* 1 |  |  |  | EU643714 |  |  | 13 |
| *Campanula lusitanica** | *Rapunculus* 1 |  |  |  |  |  | FM212715 | 3 |
| *Campanula lusitanica** | *Rapunculus* 1 |  |  |  |  |  | FM212716 | 3 |
| *Campanula lusitanica** | *Rapunculus* 1 |  |  |  |  |  | FM212717 | 3 |
| *Campanula lusitanica** | *Rapunculus* 1 |  |  |  |  |  | FM212718 | 3 |
| *Campanula lusitanica** | *Rapunculus* 1 |  |  |  |  |  | FM212720 | 3 |
| *Campanula lusitanica** | *Rapunculus* 1 |  |  |  |  |  | FM212708 | 3 |
|  |  |  | **atpB** | **matK** | **rbcL** | **trnL-F** | **ITS** |  |
| **Species** | **Clade** | **Voucher** | **Genbank** | **Genbank** | **Genbank** | **Genbank** | **Genbank** | **Source** |
| *Campanula lusitanica** | *Rapunculus* 1 |  |  |  |  |  | FM212710 | 3 |
| *Campanula lusitanica** | *Rapunculus* 1 |  |  |  |  |  | FM212721 | 3 |
| *Campanula lusitanica** | *Rapunculus* 1 |  |  |  |  |  | FM212722 | 3 |
| *Campanula lusitanica** | *Rapunculus* 1 |  |  |  |  |  | FM212723 | 3 |
| *Campanula lusitanica** | *Rapunculus* 1 |  |  |  |  |  | FM212724 | 3 |
| *Campanula lusitanica** | *Rapunculus* 1 |  |  |  |  |  | FM212725 | 3 |
| *Campanula macrostyla* | *Campanula* s. str. |  |  |  | EU643722 |  |  | 13 |
| *Campanula marchesettii* | *Rapunculus* 1 |  |  |  |  |  | DQ304612 | 11 |
| *Campanula medium* | *Campanula* s. str. |  | EU437607 | EU713272 | EU713379 |  |  | 8 |
| *Campanula medium* | *Campanula* s. str. |  |  |  | FJ587261 |  |  | 13 |
| *Campanula mirabilis* | *Campanula* s. str. |  | EU437612 | EU713277 | EU713384 |  |  | 8 |
| *Campanula mollis* | *Campanula* s. str. |  | EU437603 | EU713268 | EU713375 |  |  | 8 |
| *Campanula mollis* | *Campanula* s. str. |  |  |  | EU643721 |  |  | 13 |
| *Campanula moravica* | *Rapunculus* 1 |  |  |  |  | EF088740 |  | 12 |
| *Campanula moravica* | *Rapunculus* 1 |  |  |  | FJ587262 |  |  | 13 |
| *Campanula morettiana* | *Rapunculus* 1 |  |  |  |  |  | DQ304602 | 11 |
| *Campanula olympica* | *Rapunculus* 1 |  |  |  |  | EF088741 |  | 12 |
| *Campanula olympica* | *Rapunculus* 1 |  |  |  | FJ587263 |  |  | 13 |
| *Campanula parryi idahoensis* | *Rapunculus* 1 | WWB 22721 | JN571929 | JN571949 | JN571970 | JN572008 |  |  |
| *Campanula parryi idahoensis* | *Rapunculus* 1 | WWB 22722 | JN571930 | JN571950 | JN571971 | JN572009 | JN571993 |  |
| *Campanula parryi idahoensis* | *Rapunculus* 1 | WWB 22723 |  |  |  | JN572010 |  |  |
| *Campanula parryi parryi* | *Rapunculus* 1 | RM 779069 |  |  |  | JN572012 |  |  |
| *Campanula parryi parryi* | *Rapunculus* 1 |  | EU437675 | EU713342 | EU713449 |  |  | 8 |
| *Campanula parryi parryi* | *Rapunculus* 1 |  |  |  |  | EF213147 |  | 16 |
| *Campanula parryi parryi* | *Rapunculus* 1 | RM 749390 | JN571931 | JN571951 | JN571973 |  | JN571994 |  |
| *Campanula parryi parryi* | *Rapunculus* 1 | ASU 116235 |  | JN571952 | JN571974 | JN572011 |  |  |
| *Campanula patula* | *Rapunculus* 1 |  |  |  |  |  | FM212739 | 3 |
| *Campanula patula* | *Rapunculus* 1 |  |  |  |  | EF213148 |  | 16 |
| *Campanula patula** | *Rapunculus* 1 |  |  |  |  | EF088697 |  | 12 |
| *Campanula pelviformis* | *Campanula* s. str. |  | EU437578 | EU713243 | EU713350 |  |  | 8 |
| *Campanula pendula** | *Campanula* s. str. |  | EU437613 | EU713278 | EU713385 |  |  | 8 |
| *Campanula peregrina* | *Musschia* clade |  | EU437654 | EU713320 | EU713427 |  |  | 8 |
| *Campanula peregrina* | *Musschia* clade |  |  |  | EU643719 |  |  | 13 |
| *Campanula persicifolia* | *Rapunculus* 2 |  |  |  |  |  | AY322030, AY331443 | 5 |
| *Campanula persicifolia* | *Rapunculus* 2 |  |  |  |  | FJ426573 |  | 6 |
| *Campanula persicifolia* | *Rapunculus* 2 |  | EU437657 | EU713324 | EU713431 |  |  | 8 |
| *Campanula persicifolia* | *Rapunculus* 2 |  |  |  |  |  | DQ304590 | 11 |
| *Campanula persicifolia* | *Rapunculus* 2 |  |  |  |  | EF088743 |  | 12 |
| *Campanula persicifolia* | *Rapunculus* 2 |  |  |  | FJ587264 |  |  | 13 |
| *Campanula persicifolia* | *Rapunculus* 2 |  |  |  |  | EF213149 |  | 16 |
|  |  |  | **atpB** | **matK** | **rbcL** | **trnL-F** | **ITS** |  |
| **Species** | **Clade** | **Voucher** | **Genbank** | **Genbank** | **Genbank** | **Genbank** | **Genbank** | **Source** |
| *Campanula pinatzii* | *Campanula* s. str. |  | EU437624 | EU713289 | EU713396 |  |  | 8 |
| *Campanula pinatzii* | *Campanula* s. str. |  |  |  | FJ587265 |  |  | 13 |
| *Campanula piperi* | *Rapunculus* 1 | WWB 22724 | JN571932 | JN571953 | JN571975 | JN572013 |  |  |
| *Campanula piperi* | *Rapunculus* 1 | WWB 22725 |  |  |  | JN572014 |  |  |
| *Campanula piperi* | *Rapunculus* 1 | WWB 22726 | JN571933 | JN571954 | JN571976 | JN572015 | JN571995 |  |
| *Campanula piperi* | *Rapunculus* 1 | WWB 22727 |  |  |  | JN572016 |  |  |
| *Campanula portenschlagiana* | *Rapunculus* 1 |  |  |  |  | FJ426587 |  | 6 |
| *Campanula portenschlagiana* | *Rapunculus* 1 |  |  |  |  |  | DQ304600 | 11 |
| *Campanula poscharskyana* | *Rapunculus* 1 |  |  |  |  | FJ426588 |  | 6 |
| *Campanula poscharskyana* | *Rapunculus* 1 |  |  |  |  |  | DQ304601 | 11 |
| *Campanula poscharskyana* | *Rapunculus* 1 |  |  |  |  | EF088747 |  | 12 |
| *Campanula poscharskyana* | *Rapunculus* 1 |  |  |  | FJ587266 |  |  | 13 |
| *Campanula prenanthoides* | *Rapunculus* 2 |  |  |  |  | EF088748 |  | 12 |
| *Campanula prenanthoides* | *Rapunculus* 2 | WWB 22736 | JN571934 |  | JN571972 |  |  |  |
| *Campanula propinqua* | *Campanula* s. str. |  |  |  | FJ587267 |  |  | 13 |
| *Campanula ptarmicifolia* | *Campanula* s. str. |  |  |  | EU643710 |  |  | 13 |
| *Campanula pterocaula* | *Rapunculus* 2 |  |  |  |  | EF088751 | EF090542, EF090583 | 12 |
| *Campanula pterocaula* | *Rapunculus* 2 |  |  |  | FJ587268 |  |  | 13 |
| *Campanula pubicalyx* | *Trachelium* clade |  |  |  | EU643717 |  |  | 13 |
| *Campanula pulla* | *Rapunculus* 1 |  |  |  |  |  | DQ304605 | 11 |
| *Campanula punctata* | *Campanula* s. str. |  |  |  | EU643725 |  |  | 13 |
| *Campanula pyramidalis* | *Rapunculus* 1 |  |  |  |  |  | AY322034, AY331447 | 5 |
| *Campanula pyramidalis* | *Rapunculus* 1 |  |  | EU713322 | EU713429 |  |  | 8 |
| *Campanula pyramidalis* | *Rapunculus* 1 |  |  |  |  |  | DQ304606 | 11 |
| *Campanula pyramidalis* | *Rapunculus* 1 |  |  |  |  | EF088754 |  | 12 |
| *Campanula pyramidalis* | *Rapunculus* 1 |  |  |  |  | GQ254919 |  | 15 |
| *Campanula quercetorum* | *Campanula* s. str. |  |  |  | FJ587269 |  |  | 13 |
| *Campanula radula* | *Campanula* s. str. |  |  |  | FJ587270 |  |  | 13 |
| *Campanula raineri* | *Rapunculus* 1 |  |  |  |  |  | DQ304604 | 11 |
| *Campanula ramosissima* | *Campanula* s. str. |  |  |  | L13861 |  |  | 10 |
| *Campanula rapunculoides**† | *Campanula* s. str. |  | EU437621 | EU713286 | EU713393 |  |  | 8 |
| *Campanula rapunculoides*† | *Campanula* s. str. |  | EU437620 | EU713285 | EU713392 |  |  | 8 |
| *Campanula rapunculoides*† | *Campanula* s. str. / *Rapunculus* 1? |  |  |  | FJ587271 |  |  | 13 |
| *Campanula rapunculus* | *Rapunculus* 1 |  |  |  |  |  | FM212738 | 3 |
| *Campanula rapunculus* | *Rapunculus* 1 |  |  |  |  | EF088758 | EF090546, EF090587 | 12 |
| *Campanula rapunculus* | *Rapunculus* 1 |  |  |  | FJ587272 |  |  | 13 |
| *Campanula rapunculus** | *Rapunculus* 1 |  |  |  |  |  | DQ304609 | 11 |
| *Campanula reatina* | *Rapunculus* 1 |  |  |  |  | FJ426589 |  | 6 |
| *Campanula reatina* | *Rapunculus* 1 |  |  |  |  |  | DQ304599 | 11 |
|  |  |  | **atpB** | **matK** | **rbcL** | **trnL-F** | **ITS** |  |
| **Species** | **Clade** | **Voucher** | **Genbank** | **Genbank** | **Genbank** | **Genbank** | **Genbank** | **Source** |
| *Campanula reverchonii* | *Rapunculus* 2 |  |  |  |  |  | AY322036, AY331449 | 5 |
| *Campanula reverchonii* | *Rapunculus* 2 |  | EU437594 | EU713259 | EU713366 |  |  | 8 |
| *Campanula robinsiae* | *Rapunculus* 2 |  | EU437642 | EU713308 | EU713415 |  |  | 8 |
| *Campanula rotundifolia* | *Rapunculus* 1 |  |  |  |  |  | FM212736 | 3 |
| *Campanula rotundifolia* | *Rapunculus* 1 |  |  |  |  |  | AY322037, AY331450 | 5 |
| *Campanula rotundifolia* | *Rapunculus* 1 |  | EU437668 | EU713335 | EU713442 |  |  | 8 |
| *Campanula rotundifolia* | *Rapunculus* 1 |  |  |  |  |  | DQ304615 | 11 |
| *Campanula rotundifolia* | *Rapunculus* 1 |  |  |  |  | EF088759 |  | 12 |
| *Campanula rotundifolia* | *Rapunculus* 1 |  |  |  | FJ587273 |  |  | 13 |
| *Campanula rotundifolia* | *Rapunculus* 1 |  |  |  |  | GQ254920 |  | 15 |
| *Campanula rotundifolia* | *Rapunculus* 1 |  |  |  |  | EF213153 |  | 16 |
| *Campanula rotundifolia* | *Rapunculus* 1 | WWB 22738 | JN571935 | JN571955 | JN571977 | JN572017 | JN571988 |  |
| *Campanula rotundifolia* | *Rapunculus* 1 | WS 242584 | JN571936 | JN571957 | JN571979 |  |  |  |
| *Campanula rotundifolia* | *Rapunculus* 1 | WS 342673 | JN571937 | JN571958 | JN571980 |  |  |  |
| *Campanula rotundifolia* | *Rapunculus* 1 | WWB 5022 | JN571938 | JN571959 | JN571981 |  |  |  |
| *Campanula rotundifolia* | *Rapunculus* 1 | WWB 20359 |  | JN571956 | JN571978 |  |  |  |
| *Campanula rotundifolia* | *Rapunculus* 1 |  | EU437669 | EU713336 | EU713443 |  |  | 8 |
| *Campanula rumeliana** | *Campanula* s. str. |  | EU437619 | EU713284 | EU713391 |  |  | 8 |
| *Campanula rumeliana** | *Campanula* s. str. |  |  |  | EU643726 |  |  | 13 |
| *Campanula sarmatica* | *Campanula* s. str. |  | EU437614 | EU713279 | EU713386 |  |  | 8 |
| *Campanula saxatilis* | *Campanula* s. str. |  | EU437577 | EU713242 | EU713349 |  |  | 8 |
| *Campanula scabrella* | *Rapunculus* 1 | WWB 22728 |  |  |  | JN572018 |  |  |
| *Campanula scabrella* | *Rapunculus* 1 | WWB 22729 | JN571939 | JN571960 | JN571982 | JN572019 | JN571989 |  |
| *Campanula scabrella* | *Rapunculus* 1 | RM 408150 |  | JN571961 | JN571983 | JN572020 |  |  |
| *Campanula scabrella* | *Rapunculus* 1 | WWB 22730 |  |  |  | JN572021 |  |  |
| *Campanula scheuchzeri* | *Rapunculus* 1 |  |  |  |  |  | DQ304614 | 11 |
| *Campanula scheuchzeri* | *Rapunculus* 1 |  |  |  |  | EF088762 |  | 12 |
| *Campanula scheuchzeri* | *Rapunculus* 1 |  |  |  | FJ587275 |  |  | 13 |
| *Campanula scheuchzeri* | *Rapunculus* 1 | WS 268649 | JN571940 | JN571962 | JN571984 |  |  |  |
| *Campanula sclerotricha* | *Campanula* s. str. |  |  |  | FJ587276 |  |  | 13 |
| *Campanula scoparia* | *Campanula* s. str. |  |  |  | FJ587277 |  |  | 13 |
| *Campanula scouleri*† | *Rapunculus* 2 |  | EU437678 | EU713345 | EU713452 |  |  | 8 |
| *Campanula scouleri*† | *Rapunculus* 1 | WWB 22739 | JN571941 | JN571963 | JN571985 | JN572022 | JN571990 |  |
| *Campanula secundiflora* | *Rapunculus* 1 |  |  |  |  |  | DQ304608 | 11 |
| *Campanula secundiflora* | *Rapunculus* 1 |  |  |  |  | GQ254922 |  | 15 |
| *Campanula semisecta* | *Campanula* s. str. |  |  |  | FJ587278 |  |  | 13 |
| *Campanula sibirica* | *Campanula* s. str. |  |  |  | FJ587279 |  |  | 13 |
| *Campanula sparsa* | *Rapunculus* 1 |  |  |  |  | EF213159 |  | 16 |
| *Campanula spatulata* | *Rapunculus* 1 |  | EU437670 | EU713337 | EU713444 |  |  | 8 |
| *Campanula speciosa* | *Campanula* s. str. |  |  |  | FJ587280 |  |  | 13 |
|  |  |  | **atpB** | **matK** | **rbcL** | **trnL-F** | **ITS** |  |
| **Species** | **Clade** | **Voucher** | **Genbank** | **Genbank** | **Genbank** | **Genbank** | **Genbank** | **Source** |
| *Campanula speciosa** | *Campanula* s. str. |  |  |  | FJ587240 |  |  | 13 |
| *Campanula spicata* | *Campanula* s. str. |  |  |  | FJ587281 |  |  | 13 |
| *Campanula stenocodon* | *Rapunculus* 1 |  |  |  |  |  | DQ304620 | 11 |
| *Campanula stevenii* | *Rapunculus* 2 |  |  |  |  |  | DQ304591 | 11 |
| *Campanula stevenii* | *Rapunculus* 2 |  |  |  |  |  | AY322041, AY331454 | 5 |
| *Campanula stevenii* | *Rapunculus* 2 |  |  |  |  | EF088770 |  | 12 |
| *Campanula stevenii* | *Rapunculus* 2 |  |  |  | FJ587282 |  |  | 13 |
| *Campanula stricta* | *Campanula* s. str. |  |  |  | FJ587283 |  |  | 13 |
| *Campanula thyrsoides* | *Campanula* s. str. |  |  |  | EU643723 |  |  | 13 |
| *Campanula tommasiniana* | *Rapunculus* 1 |  |  |  |  | FJ426590 |  | 6 |
| *Campanula tommasiniana* | *Rapunculus* 1 |  |  |  |  |  | DQ304611 | 11 |
| *Campanula tommasiniana* | *Rapunculus* 1 |  |  |  |  | GQ254923 |  | 15 |
| *Campanula trachelium* | *Campanula* s. str. |  |  |  | DQ356118 |  |  | 1 |
| *Campanula trachelium* | *Campanula* s. str. |  |  |  | FJ587285 |  |  | 13 |
| *Campanula tubulosa* | *Campanula* s. str. |  | EU437580 | EU713245 | EU713352 |  |  | 8 |
| *Campanula uniflora* | *Rapunculus* 2 |  |  |  |  | FJ426574 |  | 6 |
| *Campanula uniflora* | *Rapunculus* 2 |  |  |  |  |  | DQ304588 | 11 |
| *Campanula uniflora* | *Rapunculus* 2 | ALA V134509 |  | JN571964 | JN571986 |  | JN571997 |  |
| *Campanula versicolor* | *Rapunculus* 1 |  |  |  |  | FJ426591 |  | 6 |
| *Campanula versicolor* | *Rapunculus* 1 |  |  |  |  |  | DQ304607 | 11 |
| *Campanula waldsteiniana* | *Rapunculus* 1 |  |  |  |  |  | DQ304610 | 11 |
| *Campanula waldsteiniana* | *Rapunculus* 1 |  |  |  |  | GQ254927 |  | 15 |
| *Campanula waldsteiniana* | *Rapunculus* 1 |  |  |  |  | EF213163 |  | 16 |
| *Campanula wilkinsiana* | *Rapunculus* 2 | WWB 22740 | JN571942 |  | JN571987 | JN572026 |  |  |
| *Campanula witasekiana* | *Rapunculus* 1 |  |  |  |  | EF213164 |  | 16 |
| *Canarina canariensis* | Platycodoneae |  |  |  | DQ356115 |  |  | 1 |
| *Canarina canariensis* | Platycodoneae |  | EU437581 | EU713246 | EU713353 |  |  | 8 |
| *Canarina canariensis* | Platycodoneae |  |  |  | EU643709 |  |  | 13 |
| *Codonopsis dicentrifolia* | Platycodoneae |  | EU437585 | EU713250 | EU713357 |  |  | 8 |
| *Codonopsis gracilis** | Platycodoneae |  | EU437617 | EU713282 | EU713389 |  |  | 8 |
| *Codonopsis kawakamii* | Platycodoneae |  | EU437588 | EU713253 | EU713360 |  |  | 8 |
| *Codonopsis lanceolata* | Platycodoneae |  | EU437583 | EU713248 | EU713355 |  |  | 8 |
| *Codonopsis viridis* | Platycodoneae |  |  |  | AY655148 |  |  | 4 |
| *Codonopsis viridis* | Platycodoneae |  | EU437584 | EU713249 | EU713356 |  |  | 8 |
| *Craterocapsa tarsodes* | Wahlenbergieae |  | EU437636 | EU713301 | EU713408 |  |  | 8 |
| *Cyananthus lobatus* | Platycodoneae |  |  |  | AY655149 |  |  | 4 |
| *Cyananthus lobatus* | Platycodoneae |  | EU437587 | EU713252 | EU713359 |  |  | 8 |
| *Cyphia elata* | outgroup |  | EU437599 | EU713264 | EU713371 |  |  | 8 |
| *Edraianthus graminifolius* | *Campanula* s. str. |  |  |  | AY655150 |  |  | 4 |
| *Edraianthus graminifolius* | *Campanula* s. str. |  | EU437608 | EU713273 | EU713380 |  |  | 8 |
|  |  |  | **atpB** | **matK** | **rbcL** | **trnL-F** | **ITS** |  |
| **Species** | **Clade** | **Voucher** | **Genbank** | **Genbank** | **Genbank** | **Genbank** | **Genbank** | **Source** |
| *Edraianthus graminifolius* | *Campanula* s. str. |  |  |  | EU643705 |  |  | 13 |
| *Favratia zoysii** | *Rapunculus* 1 |  |  |  |  |  | DQ304603 | 11 |
| *Feeria angustifolia*† | *Campanula* s. str. |  | EU437622 | EU713287 | EU713394 |  |  | 8 |
| *Feeria angustifolia*† | Wahlenbergieae |  |  |  | EU643711 |  |  | 13 |
| *Githopsis diffusa* | *Rapunculus* 2 |  |  |  |  |  | AY322056, AY331469 | 5 |
| *Githopsis diffusa* | *Rapunculus* 2 |  | EU437644 | EU713310 | EU713417 |  |  | 8 |
| *Githopsis pulchella* | *Rapunculus* 2 |  | EU437647 | EU713313 | EU713420 |  |  | 8 |
| *Hanabusaya asiatica* | *Rapunculus* 1 |  |  |  |  |  | AY322057, AY331470 | 5 |
| *Hanabusaya asiatica* | *Rapunculus* 1 |  | EU437658 | EU713325 | EU713432 |  |  | 8 |
| *Heterochaenia ensifolia* | Wahlenbergieae |  | EU437666 | EU713333 | EU713440 |  |  | 8 |
| *Heterocodon rariflorum* | *Rapunculus* 2 |  |  |  |  |  | AY322058, AY331471 | 5 |
| *Heterocodon rariflorum* | *Rapunculus* 2 |  | EU437641 | EU713307 | EU713414 |  |  | 8 |
| *Jasione crispa* | *Wahlenbergieae* |  | EU437618 | EU713283 | EU713390 |  |  | 8 |
| *Jasione heldreichii* | *Wahlenbergieae* |  |  |  | AY655151 |  |  | 4 |
| *Jasione heldreichii* | *Wahlenbergieae* |  | EU437616 | EU713281 | EU713388 |  |  | 8 |
| *Jasione laevis* | *Wahlenbergieae* |  | EU437615 | EU713280 | EU713387 |  |  | 8 |
| *Jasione montana* | *Wahlenbergieae* |  | EU437582 | EU713247 | EU713354 |  |  | 8 |
| *Jasione montana* | *Wahlenbergieae* |  |  |  | EU643731 |  |  | 13 |
| *Legousia falcata* | *Rapunculus* 2 |  |  |  | AY655152 |  |  | 4 |
| *Legousia falcata* | *Rapunculus* 2 |  |  |  |  |  | AY322064, AY331477 | 5 |
| *Legousia falcata* | *Rapunculus* 2 |  | EU437645 | EU713311 | EU713418 |  |  | 8 |
| *Legousia hybrida* | *Rapunculus* 2 |  | EU437660 | EU713327 | EU713434 |  |  | 8 |
| *Legousia hybrida* | *Rapunculus* 2 |  |  |  |  | EF088783 | EF090558, EF090599 | 12 |
| *Legousia hybrida* | *Rapunculus* 2 |  |  |  | EU643706 |  |  | 13 |
| *Legousia pentagonia* | *Rapunculus* 2 |  | EU437595 | EU713260 | EU713367 |  |  | 8 |
| *Legousia speculum-veneris* | *Rapunculus* 2 |  |  |  |  |  | AY322065, AY331478 | 5 |
| *Legousia speculum-veneris* | *Rapunculus* 2 |  | EU437593 | EU713258 | EU713365 |  |  | 8 |
| *Lobelia cardinalis* | outgroup |  |  |  | AY655144 |  |  | 4 |
| *Lobelia cardinalis* | outgroup |  | EU437598 | EU713263 | EU713370 |  |  | 8 |
| *Merciera tenuifolia* | Wahlenbergieae |  |  |  | AY655153 |  |  | 4 |
| *Merciera tenuifolia* | Wahlenbergieae |  | EU437630 | EU713295 | EU713402 |  |  | 8 |
| *Michauxia tchihatcheffii* | *Campanula* s. str. |  | EU437574 | EU713239 | EU713346 |  |  | 8 |
| *Michauxia tchihatcheffii* | *Campanula* s. str. |  |  |  | EU643720 |  |  | 13 |
| *Microcodon glomeratum* | Wahlenbergieae |  | EU437627 | EU713292 | EU713399 |  |  | 8 |
| *Musschia aurea* | *Musschia* clade |  |  |  | AY655154 |  |  | 4 |
| *Musschia aurea* | *Musschia* clade |  | EU437638 | EU713304 | EU713411 |  |  | 8 |
| *Nesocodon mauritianus* | Wahlenbergieae |  | EU437648 | EU713314 | EU713421 |  |  | 8 |
| *Petromarula pinnata* | *Rapunculus* 2 |  |  |  | AY655155 |  |  | 4 |
| *Petromarula pinnata* | *Rapunculus* 2 |  |  |  |  |  | AY322069, AY331482 | 5 |
| *Petromarula pinnata* | *Rapunculus* 2 |  |  |  |  | FJ426585 |  | 6 |
|  |  |  | **atpB** | **matK** | **rbcL** | **trnL-F** | **ITS** |  |
| **Species** | **Clade** | **Voucher** | **Genbank** | **Genbank** | **Genbank** | **Genbank** | **Genbank** | **Source** |
| *Petromarula pinnata* | *Rapunculus* 2 |  | EU437659 | EU713326 | EU713433 |  |  | 8 |
| *Petromarula pinnata* | *Rapunculus* 2 |  |  |  |  | EF088786 |  | 12 |
| *Physoplexis comosa* | *Rapunculus* 2 |  |  |  |  |  | AY322070, AY331483 | 5 |
| *Physoplexis comosa* | *Rapunculus* 2 |  |  |  |  | FJ426586 |  | 6 |
| *Physoplexis comosa* | *Rapunculus* 2 |  | EU437590 | EU713255 | EU713362 |  |  | 8 |
| *Phyteuma globulariifolium* | *Rapunculus* 2 |  |  |  |  | FJ426582 |  | 6 |
| *Phyteuma globulariifolium* | *Rapunculus* 2 |  |  |  |  |  | DQ304583 | 11 |
| *Phyteuma orbiculare* | *Rapunculus* 2 |  |  |  |  |  | AY322071, AY331484 | 5 |
| *Phyteuma spicatum* | *Rapunculus* 2 |  |  |  |  |  | AY322072, AY331485 | 5 |
| *Phyteuma spicatum* | *Rapunculus* 2 |  | EU437589 | EU713254 | EU713361 |  |  | 8 |
| *Phyteuma spicatum* | *Rapunculus* 2 |  |  |  |  | EF088787 |  | 12 |
| *Phyteuma spicatum* | *Rapunculus* 2 |  |  |  | EU643712 |  |  | 13 |
| *Platycodon grandiflorus* | Platycodoneae |  |  |  | AY655156 |  |  | 4 |
| *Platycodon grandiflorus* | Platycodoneae |  | EU437586 | EU713251 | EU713358 |  |  | 8 |
| *Prismatocarpus diffusus* | Wahlenbergieae |  |  |  | AY655157 |  |  | 4 |
| *Prismatocarpus diffusus* | Wahlenbergieae |  | EU437629 | EU713294 | EU713401 |  |  | 8 |
| *Prismatocarpus fruticosus* | Wahlenbergieae |  | EU437634 | EU713299 | EU713406 |  |  | 8 |
| *Prismatocarpus schlechteri* | Wahlenbergieae |  | EU437632 | EU713297 | EU713404 |  |  | 8 |
| *Prismatocarpus sessilis* | Wahlenbergieae |  | EU437631 | EU713296 | EU713403 |  |  | 8 |
| *Pseudonemacladus oppositifolius* | outgroup |  | EU437600 | EU713265 | EU713372 |  |  | 8 |
| *Rhigiophyllum squarrosum* | Wahlenbergieae |  | EU437653 | EU713319 | EU713426 |  |  | 8 |
| *Roella ciliata* | Wahlenbergieae |  |  |  | AY655158 |  |  | 4 |
| *Roella ciliata* | Wahlenbergieae |  | EU437633 | EU713298 | EU713405 |  |  | 8 |
| *Siphocodon debilis* | Wahlenbergieae |  | EU437649 | EU713315 | EU713422 |  |  | 8 |
| *Siphocodon spartioides* | Wahlenbergieae |  | EU437640 | EU713306 | EU713413 |  |  | 8 |
| *Solenopsis laurentia* | outgroup |  |  |  | DQ356134 |  |  | 1 |
| *Solenopsis minuta* | outgroup |  | EU437597 | EU713262 | EU713369 |  |  | 8 |
| *Theilera guthriei* | Wahlenbergieae |  | EU437637 | EU713302 | EU713409 |  |  | 8 |
| *Theodorovia karakuschensis** | *Campanula* s. str. |  |  |  | FJ587258 |  |  | 13 |
| *Trachelium caeruleum* | *Trachelium* clade |  | EU437661 | EU713328 | EU713435 |  |  | 8 |
| *Triodanis coloradoensis* | *Rapunculus* 2 |  | EU437592 | EU713257 | EU713364 |  |  | 8 |
| *Triodanis leptocarpa* | *Rapunculus* 2 |  |  |  |  |  | AY322079, AY331492 | 5 |
| *Triodanis perfoliata* | *Rapunculus* 2 |  |  |  | AY655160 |  |  | 4 |
| *Triodanis perfoliata* | *Rapunculus* 2 |  | EU437591 | EU713256 | EU713363 |  |  | 8 |
| *Wahlenbergia angustifolia* | Wahlenbergieae |  | EU437639 | EU713305 | EU713412 |  |  | 8 |
| *Wahlenbergia berteroi* | Wahlenbergieae |  | EU437650 | EU713316 | EU713423 |  |  | 8 |
| *Wahlenbergia gloriosa* | Wahlenbergieae |  |  |  | AY655161 |  |  | 4 |
| *Wahlenbergia gloriosa* | Wahlenbergieae |  | EU437635 | EU713300 | EU713407 |  |  | 8 |
| *Wahlenbergia hederacea* | Wahlenbergieae |  | EU437628 | EU713293 | EU713400 |  |  | 8 |
| *Wahlenbergia hederacea* | Wahlenbergieae |  |  |  | EU643708 |  |  | 13 |
|  |  |  | **atpB** | **matK** | **rbcL** | **trnL-F** | **ITS** |  |
| **Species** | **Clade** | **Voucher** | **Genbank** | **Genbank** | **Genbank** | **Genbank** | **Genbank** | **Source** |
| *Wahlenbergia linifolia* | Wahlenbergieae |  | EU437651 | EU713317 | EU713424 |  |  | 8 |
| *Wahlenbergia lobelioides* | Wahlenbergieae |  |  |  | EU643707 |  |  | 13 |

**References**

1. Antonelli, A. 2008. Higher level phylogeny and evolutionary trends in Campanulaceae subfam. Lobelioideae: molecular signal overshadows morphology. Mol. Phylogenet. Evol. 46:1-18.
2. Bremer, B., Bremer, K., Heidari, N., Erixon, P., Olmstead, R. G., Anderberg, A. A., Källersjö, M. and Barkhordarian, E. 2002. Phylogenetics of asterids based on 3 coding and 3 non-coding chloroplast DNA markers and the utility of non-coding DNA at higher taxonomic levels. Mol. Phylogenet. Evol. 24:274-301.
3. Cano-Maqueda, J., Talavera, S., Arista, M. and Catalán, P. 2008. Speciation and biogeographical history of the Campanula lusitanica complex (Campanulaceae) in the Western Mediterranean region. Taxon 57:11252-11266.
4. Cosner, M.E., Raubeson, L.A. and Jansen, R.K. 2004. Chloroplast DNA rearrangements in Campanulaceae: phylogenetic utility of highly rearranged genomes. BMC Evol. Biol. 4:27
5. Eddie, W. M., T. Shulkina, J. Gaskin, R. C. Haberle, and R. K. Jansen. 2003. Phylogeny of Campanulaceae s. str. inferred from ITS sequences of nuclear ribosomal DNA. Annals of the Missouri Botanical Garden 90:334–375.
6. Frajman, B. and Schneeweiss, G.M. 2009. A Campanulaceous fate: the Albanian stenoendemic Asyneuma comosiforme in fact belongs to isophyllous Campanula. Syst. Bot. 34:595-601.
7. Ge, S., Schaal, B.A. and Hong, D.-Y. 1997. A reevaluation of the status of Adenophora lobophylla based on ITS sequences, with reference to the utility of ITS sequence in Adenophora. Zhiwu Fenlei Xuebao 35:385-395
8. Haberle, R. C., Dang, A., Lee, T., Peñaflor, C., Cortes-Burns, H., Oestreich, A., Raubeson, L., Cellinese, N., Edwards, E. J., Kim, S.-T., Eddie, W. M. M. and Jansen, R. K. 2009. Taxonomic and biogeographic implications of a phylogenetic analysis of the Campanulaceae based on three chloroplast genes. Taxon 58:715-734.
9. Kim, Y.-D., Lee, J., Suh, Y., Lee, S., Kim, S.-H. and Jansen, R. K. 1999. Molecular evidence for the phylogenetic position of Hanabusaya asiatica Nakai (Campanulaceae), an endemic species in Korea. J. Plant Biol. 42:168-173.
10. Michaels, H. J., Scott, K. M., Olmstead, R. G., Szaro, T., Jansen, R. K. and Palmer, J. D. 1993. Interfamilial relationships of the Asteraceae: insights from rbcL sequence variation. Ann. Mo. Bot. Gard. 80:742-751.
11. Park, J.-M., Kovačić, S., Liber, Z., Eddie, W.M.M. and Schneeweiss, G.M. 2006. Phylogeny and biogeography of isophyllous species of Campanula (Campanulaceae) in the Mediterranean area. Syst. Bot. 31:862-880.
12. Roquet, C., Sáez, L., Aldasoro, J. J., Susanna, A., Alarcón, M. L. and Garcia-Jacas, N. 2008. Natural delineation, molecular phylogeny and floral evolution in Campanula. Syst. Bot. 33:203-217.
13. Roquet, C., Sanmartín, I., Garcia-Jacas, N., Sáez, L., Susanna, A., Wikström, N. and Aldasoro, J. J. 2009. Reconstructing the history of Campanulaceae with a Bayesian approach to molecular dating and dispersal–vicariance analyses. Mol. Phyl. Evol. 52:575-587.
14. Senni, K., Fujii, N., Takahashi, H., Sugawara, T. and Wakabayashi, M. 2005. Intraspecific chloroplast DNA variations of the alpine plants in Japan. Acta Phytotax. Geobot. 56:265-275.
15. Stefanović, S. and Lakušić, D. 2009. Molecular reappraisal confirms that the Campanula trichocalycina-pichleri complex belongs to Asyneuma (Campanulaceae). Botanica Serbica 33:21-31.
16. Stefanović, S., Lakušić, D., Kuzmina, M., Međedović, S., Tan, K. and Stevanović, V. 2008. Molecular phylogeny of Edraianthus (Grassy Bells; Campanulaceae) based on non-coding plastid DNA sequences. Taxon 57:452-475.
